# Molecular composition of the human primary visual cortex profiled by multimodal mass spectrometry imaging

**DOI:** 10.1007/s00429-018-1660-y

**Published:** 2018-04-10

**Authors:** Estibaliz González de San Román, Hans-Jürgen Bidmon, Milena Malisic, Iuliana Susnea, Astrid Küppers, Rene Hübbers, Andreas Wree, Volker Nischwitz, Katrin Amunts, Pitter F. Huesgen

**Affiliations:** 1 Central Institute of Engineering, Electronics and Analytics, ZEA-3, Forschungszentrum Jülich, Jülich, Germany; 2 Cécile and Oskar Vogt Institute of Brain Research, Medical Faculty, Heinrich Heine University Düsseldorf, Düsseldorf, Germany; 3 Institute of Neuroscience and Medicine, INM-1, Forschungszentrum Jülich, Jülich, Germany; 4 Institute of Anatomy, Rostock University Medical Center, Rostock, Germany

**Keywords:** Human brain, Peptides, Lipids, Elements, Mass spectrometry imaging, Primary visual cortex, MALDI mass spectrometry imaging, Laser ablation inductively coupled plasma mass spectrometry imaging

## Abstract

The primary visual cortex (area V1) is an extensively studied part of the cerebral cortex with well-characterized connectivity, cellular and molecular architecture and functions (for recent reviews see Amunts and Zilles, Neuron 88:1086–1107, 2015; Casagrande and Xu, Parallel visual pathways: a comparative perspective. The visual neurosciences, MIT Press, Cambridge, pp 494–506, 2004). In humans, V1 is defined by heavily myelinated fibers arriving from the radiatio optica that form the Gennari stripe in cortical layer IV, which is further subdivided into laminae IVa, IVb, IVcα and IVcβ. Due to this unique laminar pattern, V1 represents an excellent region to test whether multimodal mass spectrometric imaging could reveal novel biomolecular markers for a functionally relevant parcellation of the human cerebral cortex. Here we analyzed histological sections of three post-mortem brains with matrix-assisted laser desorption/ionization mass spectrometry imaging and laser ablation inductively coupled plasma mass spectrometry imaging to investigate the distribution of lipids, proteins and metals in human V1. We identified 71 peptides of 13 different proteins by in situ tandem mass spectrometry, of which 5 proteins show a differential laminar distribution pattern revealing the border between V1 and V2. High-accuracy mass measurements identified 123 lipid species, including glycerolipids, glycerophospholipids and sphingolipids, of which at least 20 showed differential distribution within V1 and V2. Specific lipids labeled not only myelinated layer IVb, but also IVa and especially IVc in a layer-specific manner, but also and clearly separated V1 from V2. Elemental imaging further showed a specific accumulation of copper in layer IV. In conclusion, multimodal mass spectrometry imaging identified novel biomolecular and elemental markers with specific laminar and inter-areal differences. We conclude that mass spectrometry imaging provides a promising new approach toward multimodal, molecule-based cortical parcellation.

**Electronic supplementary material:**

The online version of this article (10.1007/s00429-018-1660-y) contains supplementary material, which is available to authorized users.

## Introduction

Delineation of distinct functional regions of the brain is a prerequisite for a deeper understanding of brain function under both normal and pathological conditions. Traditionally, the functional parcellation of the human cerebral cortex has been addressed by cytoarchitectonic and myeloarchitectonic studies (Brodmann 1909; Von Economo and Koskinas 1925; Hubel and Wiesel 1977; Van Essen et al. 1992). More recent approaches have introduced observer-independent mapping techniques which significantly increased the number of cortical areas compared to Brodmann’s map and provided maps of areas in 3D-space (Zilles and Amunts 2010; Amunts and Zilles 2015). Such maps allowed the interpretation of in vivo studies employing, for example, functional magnetic resonance imaging (fMRI) with respect to the topography of activated networks and thus provide the basis for analyzing structure–function correlations (e.g. Rosenke et al. 2017; Eickhoff et al. 2005). In addition, human brain mapping has been greatly facilitated by specific molecular approaches including quantitative receptor autoradiography (Zilles and Amunts 2009). However, all commonly used cytochemical approaches to cortical parcellation require the selection of specific, previously known and often well-characterized molecules such as (radio-) labeled neurotransmitter receptor ligands, or antibodies towards peptides and proteins.

Over the past two decades, a variety of mass spectrometry imaging (MSI) techniques have been developed that enable simultaneous mapping of multiple proteins, peptides, lipids or elements and hence definition of molecular anatomy in a highly explorative manner (Chughtai and Heeren 2010). In principle, these techniques open new avenues for a detailed molecular parcellation of the brain. Matrix-assisted laser desorption/ionization mass spectrometry imaging (MALDI-MSI) is arguably the most versatile method among these, as it allows label-free detection of endogenous biomolecules including proteins and lipids and/or selected molecules of interest as, for example, drug compounds or their metabolites in any given tissue (Norris and Caprioli 2013; Shariatgorji et al. 2014). MALDI-MSI has been widely used to determine molecular changes associated with cancer and other pathologies in human tissue and animal models of human disease (Matsumoto et al. 2011; Dekker et al. 2015; Schubert et al. 2016; Martinez-Gardeazabal et al. 2017). In neurosciences, several studies used this technique to characterize the distribution of biomolecules such as lipids in the healthy (Veloso et al. 2011a, b; Manuel et al. 2015) and diseased human brain (Yuki et al. 2011). For example, studies of the human cerebral cortex described differences for the lipid distribution among gray and white matter (Veloso et al. 2011a,b; Lazar et al. 2013), but did not show a detailed cortical parcellation. Another study employed high-resolution imaging of lipids and proteins in the human optical nerve, and demonstrated region- and cell layer-specific distribution (Anderson et al. 2015).

Complementary to MALDI-MSI, laser ablation inductively coupled plasma mass spectrometry imaging (LA-ICP-MSI) allows measurement of elemental concentrations in a tissue in a spatially resolved manner. Similar to MALDI-MSI, LA-ICP-MSI has been used to study the distribution of elements in various animal models and human tissue samples (Becker et al. 2014; Susnea and Weiskirchen 2016) and to characterize changes in cerebral metal accumulation associated with disease, as, for example, in Wilson’s disease (Boaru et al. 2014).

Here we tested whether explorative techniques such as LA-ICP-MSI and MALDI-MSI could provide additional information about the presence of elements, proteins and lipids in the human brain. The primary visual cortex (V1, Area striata, or Brodmann area 17) is an anatomically well-characterized cortical area with a unique laminar pattern and was, therefore, chosen as a challenging test case. Our aim was to provide a proof-of-concept for MALDI-MSI of lipids and proteins and elemental imaging by LA-ICP-MSI as unbiased approaches to reveal regional and laminar characteristics of V1 at a resolution of 100 µm as afforded by standard commercial instruments.

## Materials and methods

### Chemicals and reagents

All chemicals and reagents were obtained in the highest commercially available quality. 2,5-dihydroxybenzoic acid (DHB), trifluoroacetic acid (TFA) and ammonium hydrogen carbonate were obtained from Sigma-Aldrich (Steinheim, Germany), trypsin and methanol were purchased from Thermo scientific (Dreieich, Germany) and methanol (LC–MS grade) was purchased from Thermo Fisher Scientific (Geel, Belgium), Water (LC–MS grade) was purchased from VWR (Leuven, Belgium).

### Human brain specimen

Post-mortem human brain samples were obtained at autopsy from the body donor program of the Center of Anatomy and Brain Research, Heinrich-Heine-University of Düsseldorf, Germany and the Institute of Anatomy of the University of Rostock, Germany (Table 1); the use of the brain samples had been approved by ethics committee of the Faculty of Medicine at the Heinrich-Heine-University of Düsseldorf under the study-No. 4863. Brain samples were stored at − 80 °C either as a whole frozen hemisphere or as 30–40 mm thick tissue slaps. Because the slide holder of the LTQ-XL-Orbitrap (Thermo Fisher Scientific, Bremen, Germany) accepted only slides with a maximum size of 75 × 25 mm, small blocks of cortical tissue in the region of the calcarine sulcus were cut using an oscillating saw (Aesculap Oscillant) with a blade precooled to − 70 °C. Cut tissue blocks were then warmed up to − 16 °C for further cutting on a cryostat (Leica CM3050, Bensheim, Germany) in serial sections of 10 µm that were mounted onto glass slides and stored at − 20 °C. In addition, after cutting sections for MALDI-MSI, the remaining tissue blocks were warmed to − 10 °C for cutting 30 µm thick serial sections for elemental imaging analysis. This enabled the use of alternating adjacent sections for lipid and protein analysis and for cyto- and myeloarchitectonic staining, whereas the directly following sections from the same tissue could be used for element analysis and histological evaluation in an alternating manner. In addition, all tissue sections used for peptide and lipid identification in MALDI-MSI experiments were afterwards rinsed in 70% ethanol for matrix removal and also used for nissl and myelin staining. These sections stained slightly weaker than non-imaged sections but allowed a direct overlay of images.

**Table 1:** Data on the human brain specimens used in this study

Brain	Gender	Age (years)	Topography	Post-mortem delay (h)
1	M	77	Left occipital lobe	5.0
2	F	91	Left occipital lobe	12.5
3	F	53	Left occipital lobe	4.0

### Cresyl violet (nissl) staining

For MALDI experiments, tissue sections were washed with ethanol to remove the matrix before staining. During LA-ICP-MS, the sample is completely ablated; therefore, a consecutive 30 µm section was used. Tissue sections were fixed in 4% buffered formalin for 10 min and rinsed 3 × 5 min in distilled water. Afterwards sections were stained for 20 min in cresyl violet solution prepared by mixing 15 ml of a freshly filtered 2% cresyl violet solution (Chroma, Köntgen, Germany) with 85 ml of sodium acetate buffer, pH 3.5. Sections were rinsed in distilled water and differentiated in 70% ethanol until specific staining results became visible. Afterwards sections were rapidly dehydrated in 96% and absolute ethanol placed 2× in xylol (Merck, Darmstadt) and cover slipped with DePex.

### Myelin staining

The same or consecutive sections of the tissue sections measured with MALDI and LA-ICP-MS were used. After matrix removal (see above) tissue sections were fixed in 4% buffered formalin for 10 min and rinsed 3 × 5 min in distilled water. Formalin-fixed tissue sections were rinsed in distilled water and placed in a solution containing 50 ml of pyridine and 25 ml acetic anhydride for 1 h in the dark. Sections were rinsed 3 × 5 min in distilled water and placed in an ammonium silver nitrate solution for 30 min in the dark. Sections were rinsed 3× in 1% acetic acid and then developed and processed according to Gallyas (1979), dehydrated and cover slipped.

### LA-ICP-MS measurements and data processing

For visualization of the spatial distribution of metals, 30-µm thick tissue sections were analysed using an inductively coupled plasma mass spectrometer (Agilent 7900, Agilent technologies, Japan) coupled to a laser ablation system (NWR 213, New Wave Research, Fremont, CA, USA). Following previous work (M-M et al. 2013), laser ablation was performed with a 60 µm spot size and 30 µm residual between lines making a y-pixel dimension of 90 µm. The ablated tissue material was transported into the ICP-MS through a transfer line using an Argon gas flow. The isotopes ^13^C, ^52^Cr, ^55^Mn, ^56^Fe, ^63^Cu and ^65^Cu were monitored. Data acquisition was synchronized with the laser ablation via a trigger signal. Acquired data were processed and images reconstructed using in-house developed software.

### Sample preparation for MALDI-MSI

Thin tissue sections for lipids and protein imaging were warmed up at room temperature for 2–3 h inside a sealed box with silica gel to avoid accumulation of condensing water, followed by 30 min drying in a vacuum desiccator. Lipid analysis was performed similar to the procedure described by (Jackson et al. 2005). In detail, 20 mg/ml DHB in 50% ethanol was directly applied as matrix substance and deposited in 24 layers using a spray device (Suncollect sprayer, SunChrom GmbH). The first layer was deposited at 10 µl/min, the second at 20 µl/min, the third at 25 µl/min and the following layers at 30 µl/min, resulting in the deposition of 141 ± 3 µg matrix/cm^2^. Two technical replicates were prepared for each brain specimen. Due to the *m*/*z* range limits of the Orbitrap instrument, proteins were in situ digested into peptides (Beine et al. 2016). Briefly, thawed tissue slides were washed in a series of three ethanol wash steps (70%/70%/100%), 1 min per step, with gentle agitation and dried in the vacuum desiccator for 1 h. Next, trypsin was dissolved in 20 mM ammonium bicarbonate buffer to a 0.1 µg/µl final concentration and applied for in situ digestion. The first layer was applied using a spray device at a flow rate of 5 µl/min, followed by 17 additional layers at a flow rate of 10 µl/min. Tissue sections were then incubated at saturated air humidity for 16 h at 37 °C using a homemade incubation chamber. After digestion, 30 mg/ml DHB in 50% methanol with 1% of TFA as additive was applied as matrix in 16 layers as described above, resulting in the deposition of 162 ± 4 µg/cm^2^. Three technical replicates were prepared for each brain specimen.

### MALDI-MS measurement

A MALDI LTQ- Orbitrap XL hybrid mass spectrometer (Thermo, Bremen, Germany) equipped with a nitrogen laser (*λ* = 337 nm, rep. rate = 60 Hz, spot size = 80 × 120 µm) was used for mass analysis. The instrument was externally calibrated using commercial peptide standard mixtures (ProteoMass calibration kit, Sigma) for either the normal (*m*/*z* 150–2000) or high (*m*/*z* 200–4000) mass range. Xcalibur (2.3) was used for MALDI-MSI data acquisition in positive ion mode. For lipid detection, the ion mass range was set to 400–1000 Da, with 10 laser shots per step at laser energy of 10 µJ. For detection of tryptic peptides the mass range was set to 800–4000 *m*/*z*, with the automatic gain control engaged and laser power set to 15 µJ. The target plate stepping distance was set to 100 µm or in special case 30 µm for both the *x*- and *y*-axes. The mass resolution was 100,000 (full width at half maximum at *m*/*z* 400). Mass spectral intensities were normalized to total ion current (TIC) at each pixel prior to image generation.

### MALDI-MS/MS for in situ identification of tryptic peptides

For identification of protein-derived tryptic peptides, the same slides previously used for MSI were re-analyzed at a raster step size of 200 µm using the Orbitrap mass analyzer. At each raster point, the top five most intense peaks were selected for MS/MS in a spiral raster pattern with a step size of 50 µm. MS/MS were acquired in the linear ion trap of the hybrid instrument, with normalized CID collision energy set to 50%, isolation width of precursor ions set to 4.0 Da and the mass range set to *m*/*z* 800–4000.

### MALDI data analysis

Peptide sequences were identified by matching acquired MS/MS spectra to the human UniProt proteome database (release 2015-09, 21,037 entries) using Sequest and Mascot (Matrix Science) as implemented in the software package Proteome Discoverer v 1.4.0.288 (Thermo Fisher Scientific, Waltham, MA, USA). Database searches were performed with a precursor mass tolerance of 10 ppm and a fragment mass tolerance of 0.8 Da, enzyme specificity set to trypsin with up to two missed cleavages and allowing methionine oxidation as a dynamic modification. The identification of two matching peptides was required to consider a protein as identified. Images of the identified peptides were generated in ImageQuest v 1.0.1 (Thermo Fischer Scientific Inc., Waltham, MA, USA). Lipid species were assigned by comparison of the measured molecular masses with the Lipid MAPS database (http://www.lipidmaps.org/), the Madison Metabolomics database (http://mmcd.nmrfam.wisc.edu) and previous reports (Berry et al. 2011; Fernandez et al. 2016). For assignment, a maximum of 5 ppm deviation between measured and theoretical mass was selected as the tolerance window. Due to the presence of salts in biological tissues, mass spectra will contain adducts of cationic salts, such as sodium or potassium apart from the protonated molecular ion [M + H]. For glycerolipid species, numbers (*x:y*) denote the total length and the number of double bonds of the acyl chains, while for sphingolipid species numbers indicate the length and number of double bonds of the acyl chain added to those of the attached sphing-4-enine (d18:1) or sphinganine (d18:0) base (Fahy et al. 2005). Images were generated with the software packages ImageQuest v.1.0.1 (Thermo Fisher Scientific, San Jose, CA, USA) and MSI reader v.0.09 (Robichaud et al. 2013; NC State University, North Carolina, U.S.A).

## Results

### Technical approach to complementary MALDI-MS imaging and LA-ICP-MS imaging of human V1

**Fig. 1:**
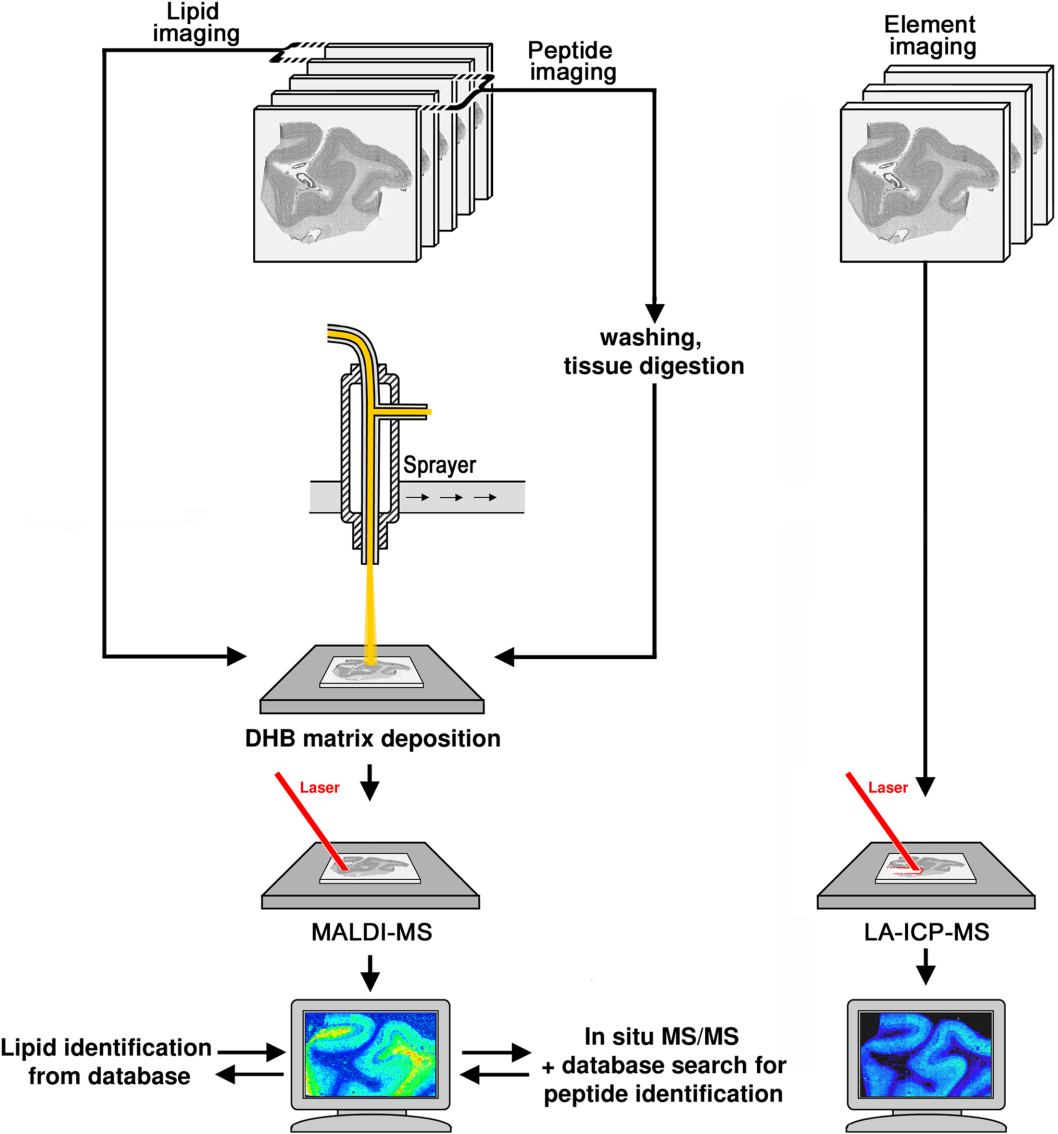
Schematic workflow. Tissue blocks containing area V1 of the human cerebral cortex were cryo-sectioned into 10-µm and 30-µm thick sections for MALDI-MSI and LA-ICP-MSI, respectively. For lipid imaging, two sections from each brain specimen were directly coated with DHB as matrix, analyzed using high-mass resolution MALDI-MS in positive mode at 100-µm lateral resolution. Molecular feature images were extracted for each *m*/*z* signal and lipids identified by comparison with lipid databases, requiring a match with a mass error < 5 ppm. For protein imaging, adjacent tissue sections were subjected to a series of washing steps to remove salts and lipids before on-tissue tryptic digestion of proteins to peptides, followed by matrix coating with DHB. MALDI-MS analysis in positive mode at 100-µm lateral resolution yielded *m*/*z* features that were visualized as intensity distribution images. For peptide identification, the top five most intense precursor ions at each spot were fragmented by MS/MS and sequences identified by database searches. For elemental imaging, cryosections were analyzed by LA-ICP-MS and images extracted for selected metals

To evaluate the utility of MALDI-MSI and LA-ICP-MS for the analysis of area V1, we first established a workflow that allowed us to analyze corresponding sections from the same specimen (Fig. 1). Both techniques require tissue sections of different thickness to be mounted on different glass object carriers with limited size. Therefore, we cut small tissue blocks of area V1 and thaw-mounted serial cryo-sections of 10 µm onto glass slides for MALDI-MSI, using consecutive tissue sections for analysis of protein-derived peptides, lipids, and histological staining. For lipid imaging, sections were directly coated with matrix and analyzed, whereas protein imaging required prior on-tissue digestion before matrix deposition due to the limited mass range of the Orbitrap mass analyzer. In MALDI-MSI studies, measurement time and data size dramatically increase with higher spatial resolution, which further negatively correlates with sensitivity (Gessel et al. 2014). Considering sample size and instrument acquisition speed, we chose to analyze three human specimens in three or two technical replicates at a moderate lateral resolution (step size) of 100 µm. The remaining adjacent tissue blocks were cut in 30 µm thick serial sections and used for element analysis by LA-ICP-MSI or histological staining. Samples were analyzed by continuous line scanning laser ablation with a focused laser beam, resulting in a horizontal resolution of 90 µm (Fig. 1).

### Imaging of tryptic peptides identifies proteins associated with specific cortical locations

**Fig. 2:**
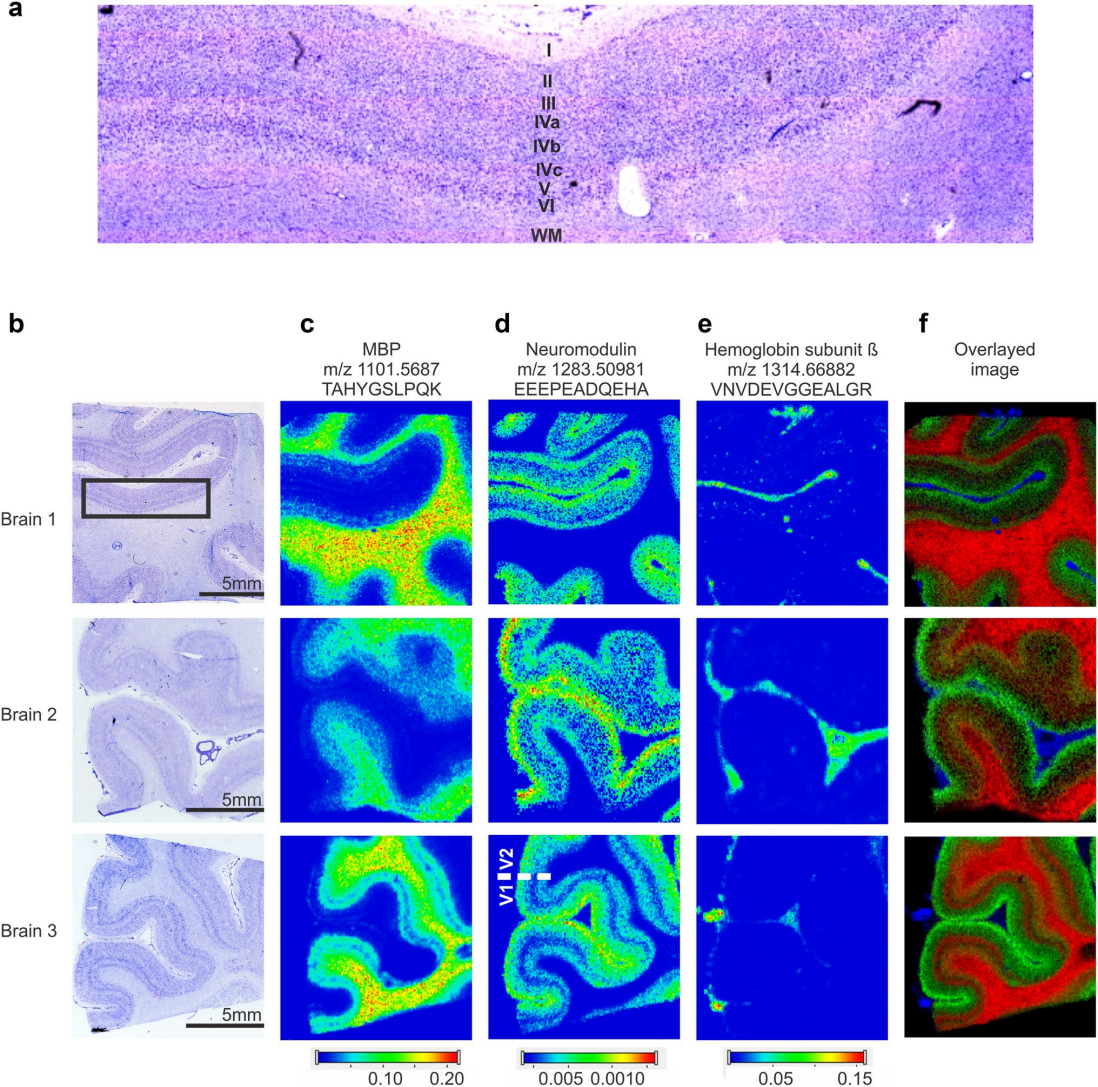
Protein distributions in the human primary visual cortex determined by MALDI-MSI. **a** High-resolution image of a Nissl-stained section. Layer I, II, III, IVa–c, V, VI and white matter are indicated. **b** Nissl-stained sections from three different post-mortem brains. **c**–**e** Molecular feature images at **c** *m*/*z* 1101.5687, identified by MS/MS as a tryptic peptide of myelin basic protein, **d** *m/z* 1283.5098, identified as tryptic peptide of neuromodulin, **e** *m*/*z* 1314.6688, identified as tryptic peptide of hemoglobin β. **f** Overlay of the three peptide images highlighting their discrete distribution, MBP as red, neuromodulin as green and hemoglobin ß as blue. Spectra were recorded in positive ion mode at 100-µm lateral resolution. Black scale bar in panel **b**: 5 mm (applies horizontally to all images of the corresponding specimen). Color scales: Peptide ion intensity in arbitrary units (applies vertically to all images of the corresponding peptide)

Employing this workflow, we identified 71 peptides from 13 different proteins by in situ MALDI-MS/MS analysis and mapped their distribution by MALDI-MSI (Supplementary Table S1, Supplementary Figs. S1-13). All of the identified proteins showed clear region- and/or cortical layer-specific distribution patterns that were reproducibly observed across all technical replicates in all three specimens. Among these, five proteins showed a differential layer-specific distribution pattern that revealed the border between V1 and V2 and thereby enabled the differentiation of these two cortical areas (Fig. 2, Supplementary Table S1, Supplementary Figs. S1-5). Sections used for MALDI-MS/MS were subsequently stained and together with adjacent sections stained for myelo- or cytoarchitecture used to further associate findings with the known cortical layers and sublayers. As expected, myelin basic protein (MBP) was observed in highest concentrations in the cortical white matter (WM), while lower concentrations were found in the infragranular cortical layers, the Gennari stripe (layer IVb) and in myelin-enriched layer I (Fig. 2b, c, Supplementary Fig. S3). Neuromodulin, also known as growth-associated protein GAP43, a protein important for neuronal pathway finding and an important component of presynaptic terminals was found in the gray matter (GM) including all cortical layers with highest concentrations in the supragranular layers (II/III, Supplementary Fig. S4). In addition neuromodulin showed very low concentrations in layer IVb and, therefore, demarcated the border between areas V1 and V2 (Fig. 2d, brain 3, Supplementary Fig. S4). In a similar manner, microtubule-associated protein tau marked this border (Supplementary Fig. S1).

In contrast to the proteins present in neural tissue, hemoglobin subunit β (HGS-β) was almost exclusively confined to intracortical blood vessels and to the intrasulcal meninges in the arachnoidea, outlining the calcarine sulcus (Fig. 2e, Supplementary Fig. S9). Overall, different protein-derived peptides showed distinct intensity gradients for GM and WM that were comparable among all three case studies (Fig. 2, Supplementary Figs. S1-13). An overlay of peptides derived from different proteins highlighted these differential expression patterns, most clearly in brain 3 (Fig. 2f).

### Element imaging shows strong compartmentalization of trace metals

**Fig. 3:**
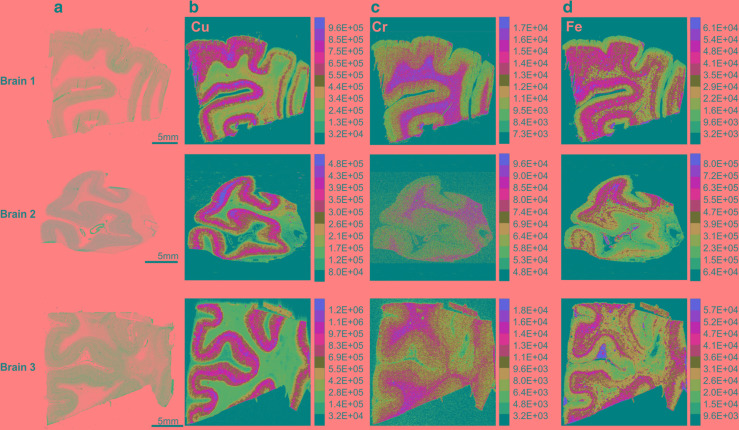
Bio-imaging of metals in area V1 of the human brain using LA-ICP-MS **a** Nissl-stained sections. **b–d** MS images obtained from monitoring ^65^Cu, ^52^Cr or ^56^Fe, respectively. Colors represent element intensity in arbitrary units, individually scaled for each image. Scale bar: 5 mm (applies to all images of the corresponding specimen)

**Fig. 4:**
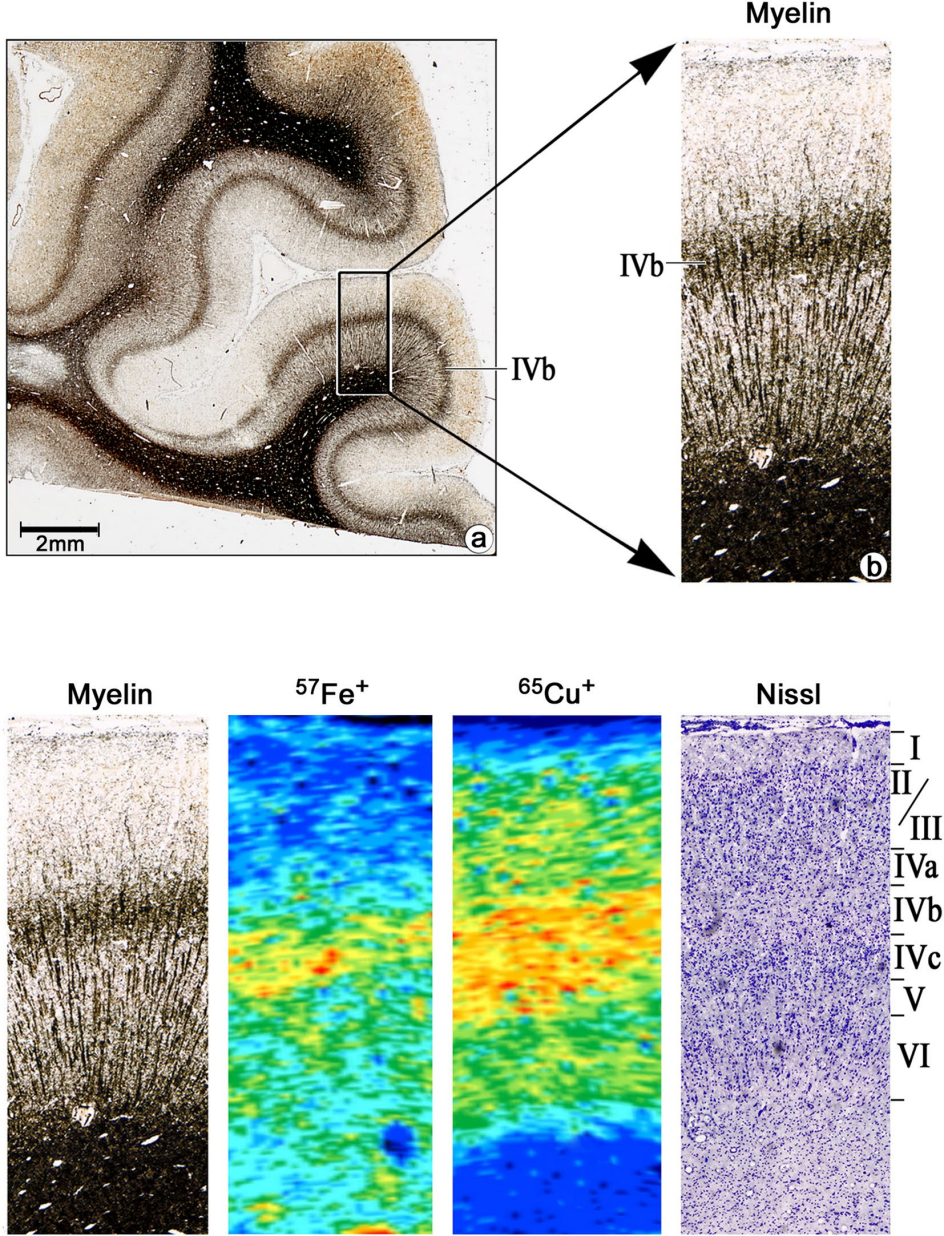
Higher magnification of a representative LA-ICP-MS image of ^65^Cu and ^57^Fe compared to cyto- and myeloarchitecture as reveal in neighboring sections of the same brain. Roman numbers indicate cortical layers; IVb serves as the major target for afferents from the lateral geniculate body. Scale bar: 2 mm

Element analysis by LA-ICP-MSI allowed the visualization of metals in human V1 (Fig. 3, Supplementary Fig. S14). Several metals, including Cu, Fe and Cr, showed strong compartmentalization (Fig. 3). For example, Cu was confined to the GM, and appeared particularly abundant in layers IVa, b and c (Fig. 3b). In contrast, concentration of Cr was high in the WM, but also in layer IVb (Fig. 3c). Accumulation of Fe was predominantly detected in blood vessels, but also in layer IV and infragranular layers (Fig. 3d). The comparison of the LA-ICP-MS images to cyto- and myeloarchitectonic images from Nissl and myelin staining, respectively, revealed that Cu was indeed confined with high concentration to layer IV (Fig. 4).

### Lipid imaging identifies markers for specific cortical layers and displays borders between cortical areas

**Fig. 5:**
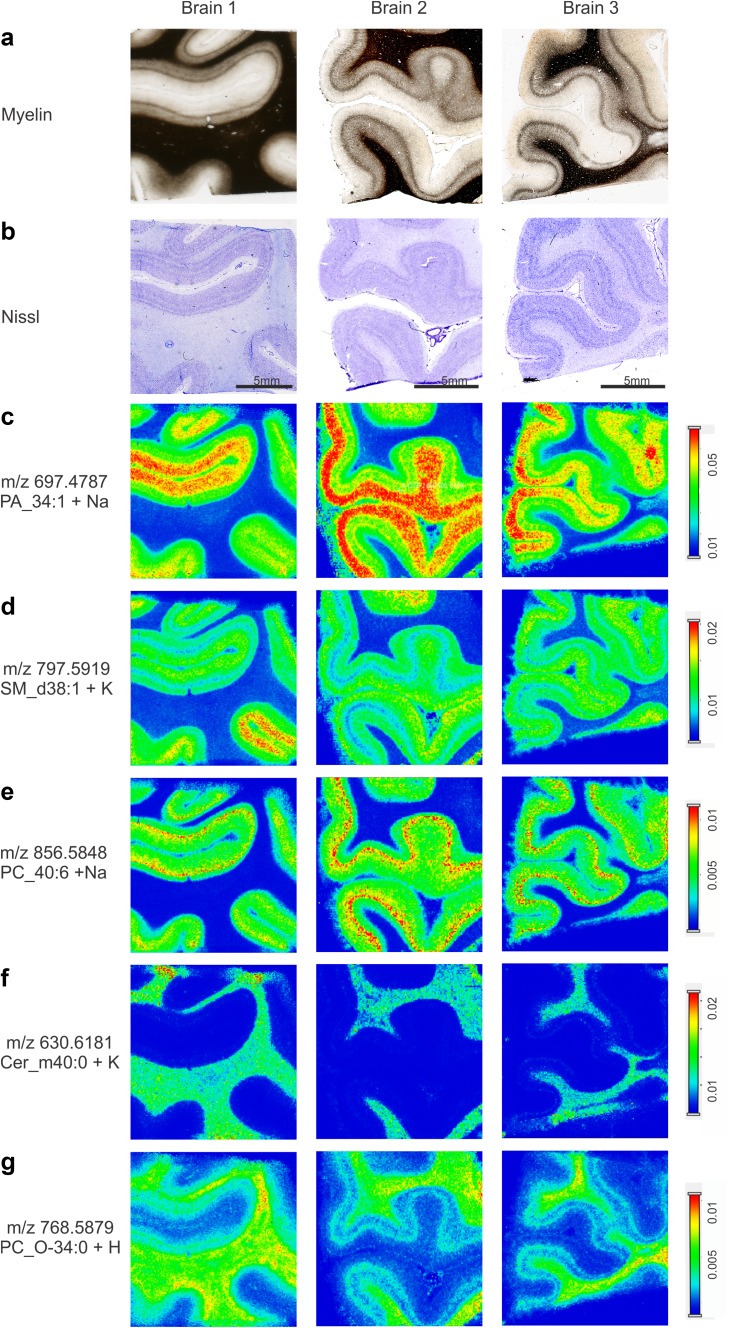
Lipid distributions in the human primary visual cortex. **a** Myelin staining. **b** Nissl staining of sections adjacent to (**a**). **c**–**g** Lipid distribution images measured by MALDI-MSI tissue sections directly adjacent to the Nissl staining shown in (**b**). Images show the distributions of ions at **c** *m*/*z* 697.4787, identified as PA_34:1 + Na, **d** *m*/*z* 797.5919, identified as SM_d38:1 + K, **e** *m*/*z* 856.5848, identified as PC_40:6 + Na, **f** *m*/*z* 630.6181, identified as Cer_m40:0 + K and **g** *m*/*z* 768.5879, identified as PC_O-34:0 + H. Images were recorded in positive ion mode at 100 µm lateral resolution. Scale bar in **b** 5 mm, applies vertically to all images of the corresponding specimen. Color bars indicate normalized lipid ion intensities (arbitrary units, applies horizontally to the corresponding lipid across all specimens)

**Fig. 6:**
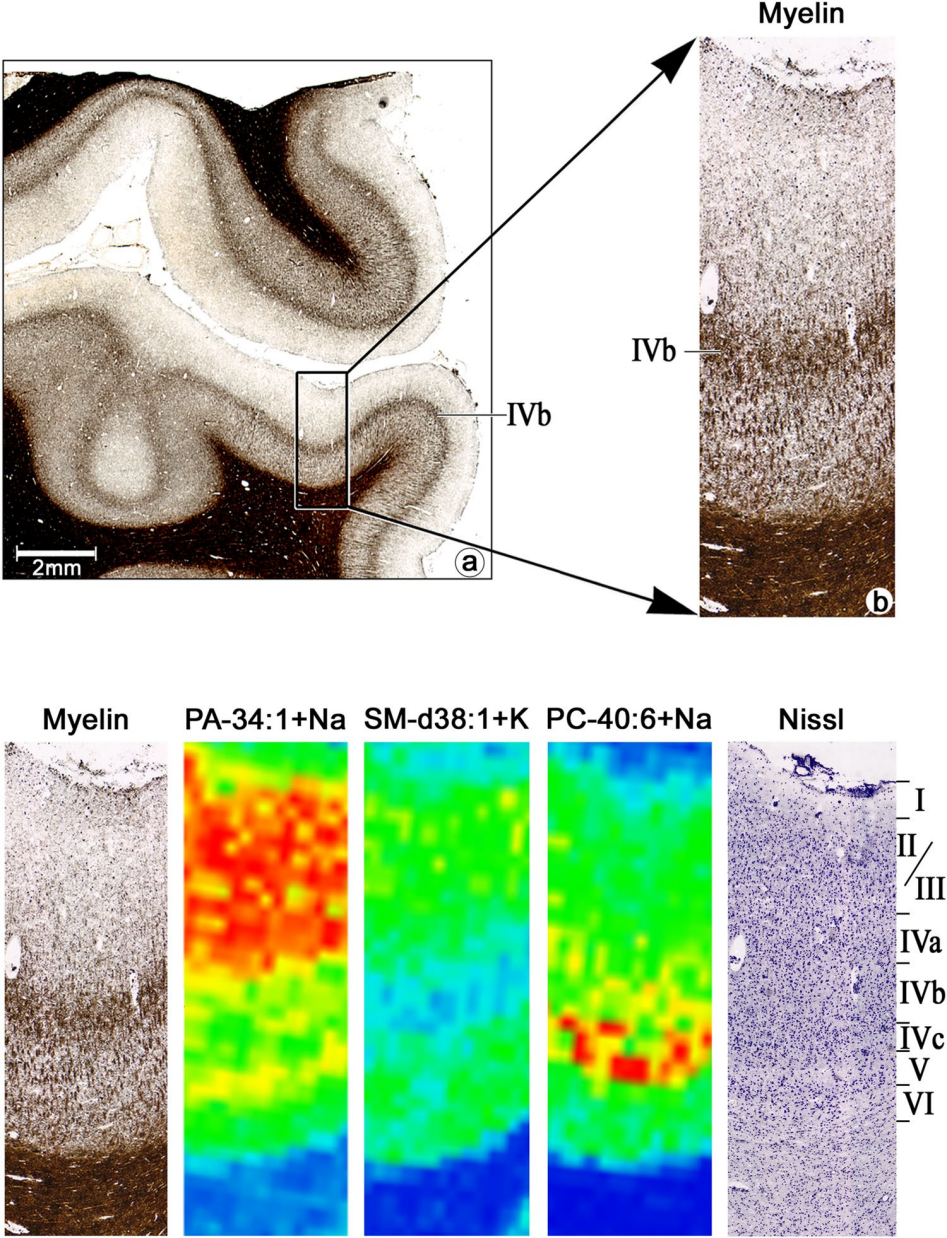
Lipid images compared to cyto- and myeloarchitecture. Note the different concentrations of the three lipids in the different cortical layers: while PA-34:0 + H shows maximal concentration in the supragranular layers, PC-40:6 + Na reaches maximal values in layer IVc; SM-d38:1 + K has minimal values in layer IVb, and medium concentrations in supra—and infragranular layers. For all three lipids, the concentrations drop down in the white matter, indicating that the lipids are associated to neuronal and/or glial cells. The images are from the brain 2. Scale bar: 2 mm

MALDI-MSI in positive ion mode revealed 123 lipid species of three different lipid subtypes such as glycerolipids, glycerophospholipids and sphingolipids (Supplementary Table S2, Supplementary Figs. S15-18). Of these, at least 20 showed a distinct laminar distribution pattern throughout V1 and clearly demarcated the border between V1 and V2 (Table 2, Supplementary Figure S15). The comparison with cyto- and myeloarchitectonic sections (Fig. 5a, b) showed that the ion at *m*/*z* 697.4787, identified as sodium adduct ion of phosphatidic acid (PA) 34:1, accumulated differentially among cortical layers (Fig. 5c). The highest abundance was observed in the supragranular layers (Fig. 5c). The lipid was also present in layer IVa (Fig. 6). The ion at *m*/*z* 797.5919, identified as potassium adduct ion of sphingomyelin (SM) d38:1, was localized in supragranular and infragranular layers with a characteristic reduction in layer IV (Fig. 5d). At higher magnification, SM_d38:1 was confined to sublayers IVa and IVc, sparing sublayer IVb completely (Fig. 6). In contrast, the ion at *m*/*z* 856.5848, identified as sodium adduct ion of phosphatidylcholine (PC)_40:6 was associated with GM and showed enhanced levels in layer IV (Fig. 5e), in particular in layer IVc (Fig. 6). The ion at *m*/*z* 630.6181, the potassium adduct of ceramide (Cer)_m40:0, was enhanced along WM, but absent or reduced to non- detectable amounts in layer IVb (Fig. 5g). In contrast, the ion at *m*/*z* 768.5879, the protonated ion of PC_O-34:0, was abundant in WM including layer IVb (Fig. 5f). In summary, we were able to identify lipids specifically accumulating in different cortical layers and even sublayers of V1 or the WM.

**Table 2:** Lipids with differential distribution pattern in V1 and V2 as determined by MALDI-MSI. Lipids were identified by comparison of the experimentally determined high-accuracy mass and their theorical *m*/*z* value

Experimental *m*/*z*	Lipid	Theoretical *m*/*z*	Error (ppm)
709.5139	[PA_O-36:2 + Na]+[/PA_P-36:1 + Na]+	709.5143	0.56
776.5906	[HexCer_d38:2 + Na]+	776.5928	2.83
778.6082	[CerP_d44:2 + Na]+	778.6085	0.38
785.4502	[PA_40:7 + K]+	785.4518	2.03
797.5926	[SM_d38:1 + K]+	797.5933	0.87
828.5503	[PC_38:6 + Na]+	828.5514	1.30
832.5819	[PC_38:4 + Na]+	832.5827	0.96
838.617	[PICer_d38:0 + H]	838.6168	− 0.23
844.5252	[PC_38:6 + K]+	844.5253	0.11
848.6368	[GlcCer_d42:2 + K]+	848.6375	0.82
851.6387	[SM_d42:2 + K]+	851.6403	1.87
856.581	[PC_40:6 + Na]+	856.5827	1.98
864.6328	[PI-Cer_d40:1 + H]	864.6324	− 0.46
865.6544	[SM_d43:2 + K]+	865.6559	1.73
866.6465	[PI-Cer_d40:0 + H]	866.6481	1.84
870.5394	[PC_40:7 + K]+	870.541	1.83
872.5567	[PC_40:6 + K]+	872.5566	− 0.11
876.6693	[GlcCer_d44:2 + K]+	876.6689	− 0.45
879.6709	[SM_d44:2 + K]+	879.6716	0.79
896.4821	[PS_42:9 + K]+	896.4838	1.89

*Cer* ceramide, *GlcCer* glucosylceramide, *PC* phosphatidylcholine, *PA* phosphatidic acid, *PS* phosphatidylserine, *SM* sphingomyelin, *PI-Cer* ceramide phosphoinositol

**Fig. 7:**
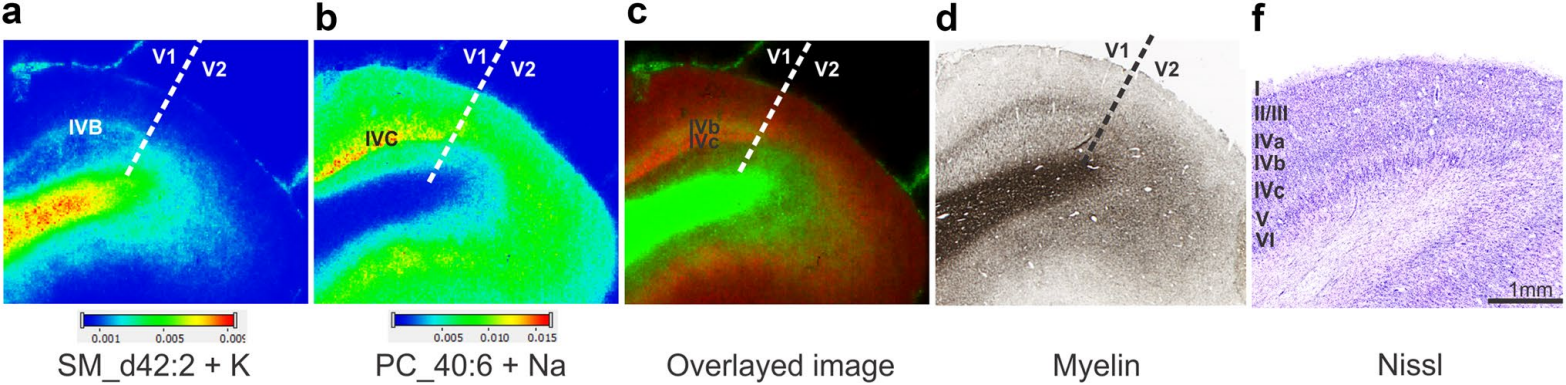
Area-specific lipid distributions in the human visual cortex reveal the border between area V1 and the secondary visual cortex, area V2. **a** The ion at *m*/*z* 851.6387 identified as SM_d42:2 increases in layer IVb (Gennari stripe) and infragranular layers at the transition of V1 and V2. **b** The distribution of ion at 856.581 identified as PC_40:6 also indicates the border of V1 and V2 by a decrease in layer IVc when moving from V1 to V2. **c** Overlayed images of SM_d42:2 + K (green) and PC_40:6 + Na+ (red) reveals distinct distribution in different layers. **d** Myelin-stained tissue section showing the border between V1 and V2. **f** Nissl-stained tissue section with layer I, II, III, IVa–c, V, VI. The spectra were recorded in positive ion mode at 30-µm lateral resolution. The images are from the brain 2. Scale bar: 1 mm (applies to all panels)

To clarify if and how lipids differed between V1 and neighboring area V2, a tissue section containing the border between V1 and V2 was measured with the highest spatial resolution possible of 30 µm. Two lipid ions, the potassium adduct ion of SM_d42:2 (Fig. 7a) and the sodium adduct ion of PC_40:6 (Fig. 7b), clearly demarcated the border of V1 and V2. A high concentration of SM_d42:2, was found in layer IVb of area V1, but disappeared when moving to V2 (Fig. 7a, d). At the same time PC_40:6 seemed to be specific of cortical layer IV, and also disappeared at the border to V2 (Fig. 5b). As visualized in an overlay of the images (Fig. 7c), SM_d42:2 specifically labeled sublayer IVb, whereas PC_40:6 was confined to IVc. Thus, several lipids distinguished the neighboring cortical areas V1 and V2 based on differences in their laminar concentration.

## Discussion

In the last decade, MSI techniques have been extensively used to study the distribution of different elements, drug compounds and endogenous biomolecules such as lipids, peptides, and proteins in various tissues (Becker et al. 2010; Shariatgorji et al. 2014; Mathur et al. 2009). The aim of the current study was to test whether commercial MALDI-MSI and LA-ICP-MSI setups would allow the identification of cortical areas in human brain sections based on element and biomolecule distribution patterns, using V1 as a region that is particularly well characterized by microanatomical and functional means (Hubel and Wiesel 1977; Hinds et al. 2009; Zilles et al. 2009; Palomero-Gallagher and Zilles 2017).

### Protein distribution patterns

In situ MS/MS analysis identified 71 peptides from 13 proteins. Many additional peptide signatures were observed, but not identified due to their low concentration, which precluded acquisition of high-quality MS/MS fragmentation spectra in situ. Although peptide identifications can be increased by parallel processing of adjacent tissue sections, followed by peptide extraction and analysis by nano-LC–MS/MS (Heijs et al. 2015), we did not follow this approach because the limited dataset already contained distinct protein/peptide patterns that were reproducibly observed in each biological and technical replicate. However, it should be noted that this does not hold true at the level of normalized intensity due to a variety of biological (differing age, gender and unknown case history; Table 1) and technical factors (different post-mortem delays, variability in tryptic digest efficiency).

Nevertheless, the peptide patterns showed a well-defined laminar resolution. Peptides derived from MBP were found in the WM and in layers IVb and I, as previously described (Horton and Hocking 1997). Layer IVb is an unequivocal characteristic of V1 that is absent in V2, MBP peptides therefore clearly demarcated V1. In contrast, neuromodulin (GAP43, Van Lookeren Campagne et al. 1989; Leu et al. 2010; Holahan 2017) and microtubule-associated protein tau demarcated layer IVb and, therefore, also V1 due to their lowest laminar concentration in this sublayer. Synapsin, a protein linked to synaptic transmission, was mainly enriched in supragranular layers, barely detected in layer IV, and then slightly increased again in the infragranular layers. This distribution was in accordance with previous studies reporting that thalamocortical terminals arriving in layer IV, which provide the driving input to V1 from the lateral geniculate nucleus, do not use synapsin (Owe et al. 2013). Furthermore, similar distribution patterns were observed for myristoylated alanine-rich C kinase substrate (MARCKS) and neuromodulin. These two presynaptic proteins, which are both involved in regulating the dynamics of the actin cytoskeleton at the synaptic membrane (Laux et al. 2000) and are considered of diagnostic value for neurodegenerative diseases (Remnestal et al. 2016), showed a distinct absence in layer IV and, therefore, differentiated V1 from V2. Both MARCKS mRNA and neuromodulin mRNA were co-expressed in monkey V1 (Higo et al. 2002, 2004). However, intense signals in layers IVb, V and VI were only observed for MARCKS (Higo et al. 2002). In contrast, we observed both proteins in layers V and VI (Supplementary Figs. S8, S11). Neurofilament light protein was predominantly observed in the GM, sparing supragranular layers, in agreement with a mesh-like distribution in layer IVa as described by immunohistochemistry (Preuss et al. 1999). The glial fibrillary acidic protein (GFAP), a protein mainly expressed in astrocytes, was mainly confined to layer I although immunohistochemical studies have shown that GFAP-positive astrocytes accumulate more in supragranular layers than in infragranular layers and WM, and that highest GFAP expression occurs in those cells forming the glia limitans (Eilam et al. 2016). It is possible that the concentration of GFAP in layers II/III is so much lower compared to layer I (or astrocytes forming the glia limitans) that it did not reach the detection limit of our method.

Overall, MALDI-MSI data showed good agreement with the distribution patterns of proteins previously studied in V1. Moreover, we identified peptide signals derived from brain acidic soluble protein (BASP-1), actin cytoplasmic protein and stathmin, all of which were previously not described in the primary visual cortex. BASP-1, a presynaptic protein involved in several cellular processes e.g. during brain development in rodents (Kropotova et al. 2013), was most abundant in supragranular layers including layer IV and is missing in layer IVb, which also made it possible to differentiate V1 from V2. Stathmin, a protein involved in regulating cytoskeletal dynamics and adult neurogenesis (Kedracka-Krok et al. 2016; Martel et al. 2016), was detected in supragranular layers and showed lower intensities in infragranular layers while sparing layer IVb completely.

Bauernfeind et al. (2015) recently studied protein distribution in different cortical areas, including cingulate cortex, motor cortex or primary visual cortex, and observed similar patterns of protein expression among supra- and infragranular layers of neocortex that were consistent with the cytoarchitectonic features independent of the region. Remarkably, no distinct signature of V1 was reported, whereas we observed distinct differences between V1 and V2 due to differential protein accumulation particularly in layer IVb. This may be explained by the different sets of proteins detected by the different technical approaches employed: Bauernfeind et al. (2015) analyzed distinct matrix spots with 200 µm diameter and detected intact proteins in a mass range between 2 and 40 kDa using an MALDI-TOF mass spectrometer, whereas we detected peptides after in situ protein digest and coating of the entire sections with a thin layer of matrix and thereby indirectly observed also proteins with a higher molecular weight, with little overlap between the two datasets. Finally, in comparison with immunohistochemical approaches where antibody cross-reactivity is hard to exclude, our in situ MS/MS analysis provided direct evidence for the sequence of the visualized peptides.

### Metal distribution patterns

Metal ion homeostasis is severely affected in a variety of neurodegenerative diseases, including Alzheimer's disease, Parkinson's disease (Bourassa and Miller 2012) and traumatic brain injury (Portbury et al. 2016). Specifically affected cortical areas can be identified by LA-ICP-MSI, which measures concentration of metals and other elements in a spatially resolved manner (Sussulini et al. 2017). Such findings make it necessary to study the distribution of metals in a more systematic way in different regions of brains of healthy controls. Here we mapped the element distributions in area V1. Discrete patterns for selected elements were found. For example, copper was distributed across the cortical cross-section, and appeared enriched in layer IV (Fig. 3). This pattern was similar to that described for Cu in the human insular cortex (Dobrowolska et al. 2008) and in agreement with previous reports indicating that Cu was more abundant in GM than WM in nonhuman primate brain (Bonilla et al. 1984; Ramos et al. 2014; Knauer et al. 2017). Iron was observed in blood vessels, as expected due to its well-known association with hemoglobin, but also along layer IV and infragranular layers. Key proteins of iron homeostasis are also involved in Cu regulation (Mueller et al. 2009), which may explain the similar distributions of Fe and Cu in layer IV and infragranular layers.

### Patterns of lipid distribution

Lipids represent the largest component of the brain (Li et al. 2017). Not only the amount, but also the number of cerebral lipid species increased along the phylogenetic line from mice over apes to man, as well as with age within a single species (Bozek et al. 2015; Li et al. 2017). This suggests that lipids might offer a key to improve our understanding of the connectome and higher cognitive functions that evolved in primates (Bozek et al. 2015; Li et al. 2017). For example, the composition of phospholipids (PLs) with varying acyl chain length and the number of unsaturated C=C double bonds define the characteristics and functional efficacy of neural membranes (Van Meer et al. 2008). Apart from their structural role, polyunsaturated lipids are further precursors for important second messengers such as arachidonic (C20:4), eicosapentaenoic (C20:5), docosapentaenoic (C22:5) and docosahexaenoic (C22:6) acids (Guichardant et al. 2011) which are partly known to be involved in neuronal signaling (Gantz and Bean 2017). This is in agreement with the recently proposed hypothesis of “small molecule co-transmission” (Nusbaum et al. 2017), which suggests that membrane compounds regulate neurotransmitter signaling independently and in conjunction transmitter receptors. Furthermore, lipid rafts have been identified as important mediators of mGlu1 receptor-mediated signaling (Roh et al. 2014). It is, therefore, of great interest to obtain detailed information on the distribution of lipids in the brain, and to correlate their occurrence with the role of these areas in certain functional networks. Mass spectrometry-based methods such as MALDI-MSI, DESI-MS, SIMS, nanoparticlelaser desorption ionization or 40 keV argon cluster SIMS (Skraskova et al. 2015; Mohammadi et al. 2016; Bodzon-Kulakowska et al. 2017) are uniquely suited to obtain such data as it is generally not possible to use techniques such as fluorescence tags and fluorescence microscopy to define the location of the relatively small and dynamic lipids.

In this study, we revealed unique regional distribution patterns of more than 120 lipid species based on high-accuracy mass measurements. Specific lipid species were found in either GM or WM, enriched in distinct cortical layers or sublayers. Among all observed lipids, we found 20 species with a relevant distribution demarcating the border between V1 and V2, mostly based on a distinct sublayer-specific localization. For instance, PC O-34:0 was highly enriched in subcortical WM, whereas PC-40:6 was neither observed in WM nor in supragranular GM. This may indicate specific function(s) confined to layer IVc, a cortical sublayer of V1 that also showed specific protein expression related to ocular dominance columns in primate experiments (Ataman et al. 2016).

PCs are the most abundant lipids in the occipital cortex, at a concentration of 19 µmol/g tissue (Abbott et al. 2013). We identified 23 different PC species, each with distinct distribution patterns. Several PCs were specifically observed in layer IVc, including PC_40:6, PC_40:7 and PC_38:6 (Figs. 5, 6; Table 2, Supplementary Fig. S15). These PCs contain the long-chain polyunsaturated docosahexaenoic acid (DHA) as FA, which is known to play an important role within the visual system, in neurotransmission at synapses and during brain development (Sugiura et al. 2009; Sugiura and Setou 2009). Furthermore, these lipids are critical for the maturation of visual functions (Uauy et al. 2001). Interestingly, fibers arriving with the Radiatio optica from the lateral geniculate nucleus via layer IVb terminate with synaptic contacts mainly at layer IVc (Casagrande and Xu 2004). Specific accumulation of polyunsaturated PCs in this region may indicate an important role within the cell membranes contributing to synaptic transmission. Consistent with that hypothesis, a loss of PC_40:6 and PC_40:7 has been observed in the parieto-occipital cortex of Parkinson patients suffering from GM atrophy and visual hallucinations (Cheng et al. 2011). Moreover, saturated PC’s such as PC_30:0, PC_32:0, PC_33:0 or PC_34:0 were found in the GM and specifically enriched in supragranular layers. The latter contains high numbers of neurons and dendrites and also higher amounts of palmitic acid (16:0) than WM (Skinner et al. 1993; Sugiura and Setou 2009; Veloso et al. 2011a, b; Martinez-Gardeazabal et al. 2017). In contrast, PCs containing 18:0, 18:1 or 18:2 as FAs are located in WM, for example, PC_33:2, PC_36:2, PC_36:1 or PC_38:2. This is in agreement with reports that showed 18:1 FA accumulation in myelin sheets (Kishimoto et al. 1969; Veloso et al. 2011a, b).

Similar to PCs, PLs containing palmitic acid, such as PA_32:1 or PA_34:1, were enriched in supragranular layers. Likewise, a PA with polyunsaturated FA, PA_40:7, accumulated specifically in layer IVc. PS_40:6, which is the most abundant PS in brain (Hicks et al. 2006), and PS_42:9 were observed along the GM, but not in layer IVc. The vast majority of the sphingolipids that we have detected are distributed along the WM and blood vessels exceptions are, for example, SM_d33:1 and SM_d38:1. The former is specifically accumulating in the supragranular layers, whereas the latter is distributed along the GM except in layer IVb. It has been reported that SM_d38:1 is decreased in Alzheimer disease in the prefrontal cortex (Chan et al. 2012). Glucosylceramide (GlcCer), which are present in higher concentration in adults compared to infant brain (Li et al. 2017), were mainly present in the WM. Also all other lipid classes, e.g. glycerolipids, showed specific distribution patterns with enhanced or reduced amounts confined to specific cortical layers. However, the function(s) of these lipids still need to be established.

### MALDI imaging of lipids resolves borders between cortical areas

Some of the tissue blocks contained not only area V1, but also parts of neighboring area V2. The border could be clearly identified by lipid biomarkers. To investigate this intricate feature in more detail, we performed additional experiments at increased resolution of 30 µm, and found that certain lipids were associated specifically with WM and the Gennari stripe (IVb), while others were specifically confined to layer IVc. For example, PC_40:6 clearly changed the distribution pattern at border V1/V2. Therefore, the human lipidome represents a new modality for functional cortical parcellation at the sublaminar level. Further studies are warranted to explore to which degree this will be applicable for other cortical and subcortical regions as well.

## Conclusions

LA-ICP-MSI and MALDI-MSI were employed for mapping element, protein and lipid distribution in area V1 of the human brain. In most cases, regional and laminar distribution patterns faithfully reflected the well-known cytoarchitectonic features of V1 which was further verified by both cell body and myelin staining. In addition, selected lipids appeared associated with cells confined to specific cortical layers. This demonstrates that multimodal proteomics, lipidomics and metallomics analysis with highly explorative MSI techniques reveals molecular markers for human brain mapping, independent of prior knowledge, target-specific reagents and at high resolution close to the cellular level. The region-specific distribution patterns of these molecules are comparable to those observed by comprehensive comparative studies of ligand binding sites for neurotransmitter receptors among functional cortical areas (e.g. Palomero-Gallagher and Zilles 2017). Both MALDI-MS and LA-ICP-MS imaging techniques still suffer from limitations such as lower spatial resolution compared to histochemistry, lower sensitivity compared to liquid mass spectrometry and necessary practical compromises between resolution, sensitivity and required measurement time (Gessel et al. 2014). However, technical (Kompauer et al. 2017) and computational (Van de Plas et al. 2015) advances already allow accelerated measurements and enabled dramatically improved lateral resolution. It should be noted that individual differences observed in metal, lipid and protein concentration among the three brain specimens studied here might be associated with different age, relate to different pre-mortem conditions or differential post-mortem handling such as delay until freezing, or differences in trypsin digest efficiency. The distribution patterns should, therefore, only be compared qualitatively, as a quantitative comparison would require higher sample numbers.

Lipids play important, but still incompletely understood roles in brain evolution and higher cognitive functions (Bozek et al. 2015; Li et al. 2017). MALDI-MSI currently offers the only option to elucidate the cerebral distribution of lipids as key determinants of cell membrane fluidity and dynamics and is, therefore, poised to play a crucial role in their integration in functional brain models (Zhao et al. 2015; Glasser et al. 2016). Furthermore, MALDI-MSI may provide a key to test the new “Small molecule co-transmission” hypothesis (Nusbaum et al. 2017) in greater detail and in a region-specific manner and thereby open avenues for a comprehensive understanding of neurotransmission-related processes and receptor trafficking.

## Electronic supplementary material

Below is the link to the electronic supplementary material.

Supplementary material 1 (DOCX 10200 KB)

## Abbreviations

V1: Primary visual cortex
V2: Secondary visual cortex
MALDI-MSI: Matrix-assisted laser desorption/ionization mass spectrometry imaging
LA-ICP-MS: Laser ablation inductively coupled plasma mass spectrometry
GM: Gray matter
WM: White matter
DHB: 2, 5-dihydroxybenzoic acid
TFA: Trifluoroacetic acid
Cer: Ceramide
DG: Diglyceride
GlcCer: Glucosylceramide
LPC: Lysophosphatidylcholine
PC: Phosphatidylcholine
PA: Phosphatidic acid
PE: Phosphatidylethanolamine
PS: Phosphatidylserine
SM: Sphingomyelin
PI-Cer: Ceramide phosphoinositol
