# Supplementary material for: Molecular composition of the human primary visual cortex profiled by multimodal mass spectrometry imaging
Source: Brain Struct Funct. 2018 Apr 10;223(6):2767–83. doi: 10.1007/s00429-018-1660-y (PMC5995978; doi:10.1007/s00429-018-1660-y)
Supplement: Supplementary file 1 — Supplementary material 1 (DOCX 10200 KB) [file 429_2018_1660_MOESM1_ESM.docx]

**SUPPLEMENTARY MATERIAL**

**Table S1.** Summary of proteins identified by in-situ MALDI-MS/MS. Proteins highlighted in red show a distinct presence or absence in layer IV and thereby visualize the border between V1 and V2.

| **UniProt ID** | **Protein** | **Identified peptides** | **Segregation of V1 and V2** | **Figure** |
| --- | --- | --- | --- | --- |
| P08073 | Brain acid soluble protein (BASP) | 8 | + | S1 |
| P17677 | Neuromodulin (GAP-43) | 4 | + | S2 |
| P10636 | Microtubule associated protein Tau (MAPT) | 3 | + | S3 |
| P02686 | Myelin basic protein (MBP) | 18 | + | S4 |
| P16949 | Stathmin | 3 | + | S5 |
| P60709 | Actin cytoplasmic | 3 | - | S6 |
| P14136 | Glial fibrillary acidic protein (GFAP) | 5 | - | S7 |
| P69905 | Hemoglobin, alpha subunit | 3 | - | S8 |
| P6871 | Hemoglobin, beta subunit | 9 | - | S9 |
| P29966 | Myristolated alanine rich C kinase substrate (MARCKS) | 5 | - | S10 |
| P07196 | Neurofilament L protein | 3 | - | S11 |
| Q92686 | Neurogranin | 2 | - | S12 |
| P17600 | Synapsin | 5 | - | S13 |
| **Total peptides** |  | **71** |  |  |
|  |  |  |  |  |


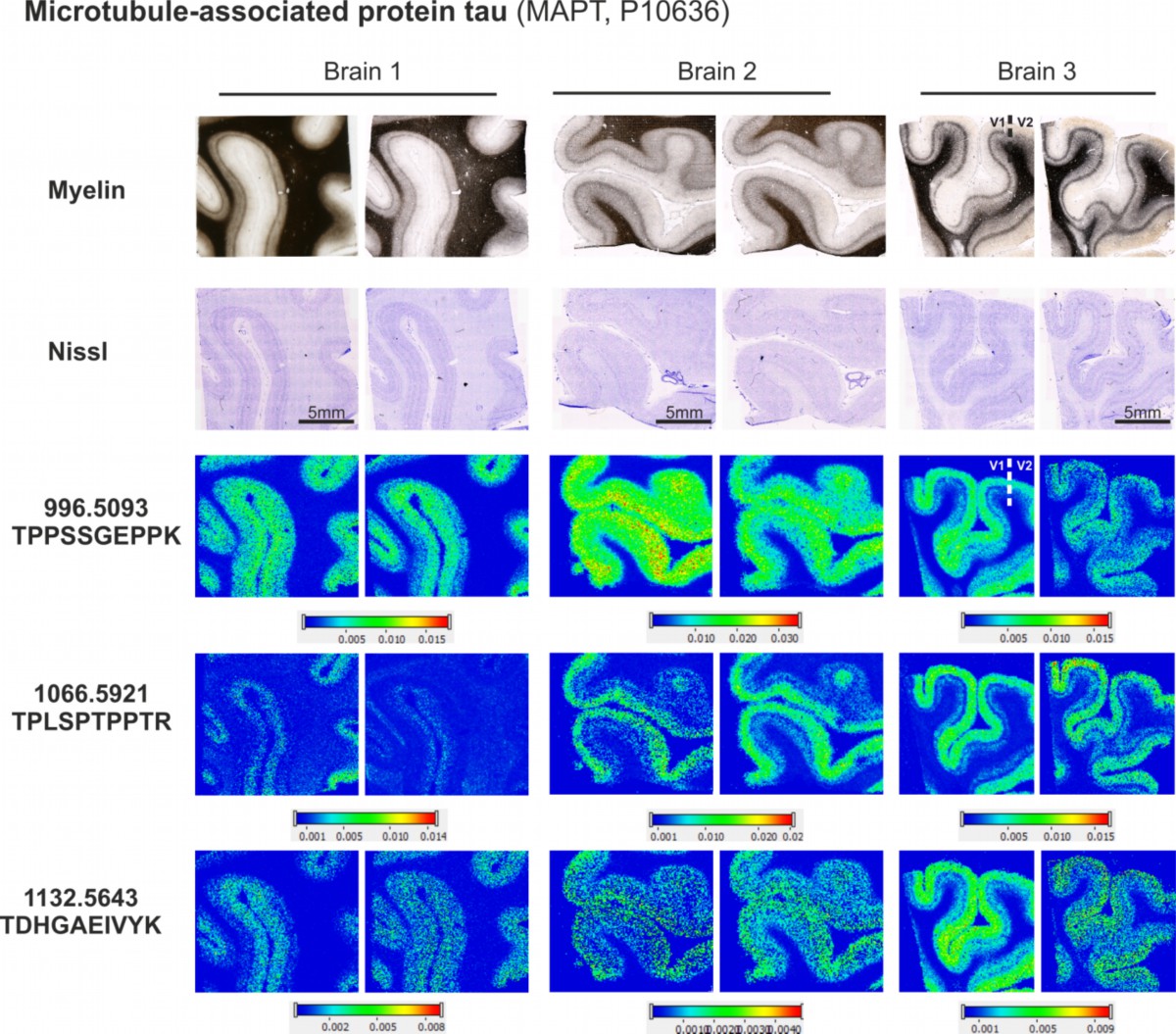


**Figure S1**. Anatomical distribution of tryptic peptides of microtubule-associated protein tau (MAPT) in human V1, identified by MALDI-MS/MS after on tissue tryptic digest. A distinct absence in layer IV defines the border V1 and V2. Two technical replicates were performed for each of the three brain specimen.


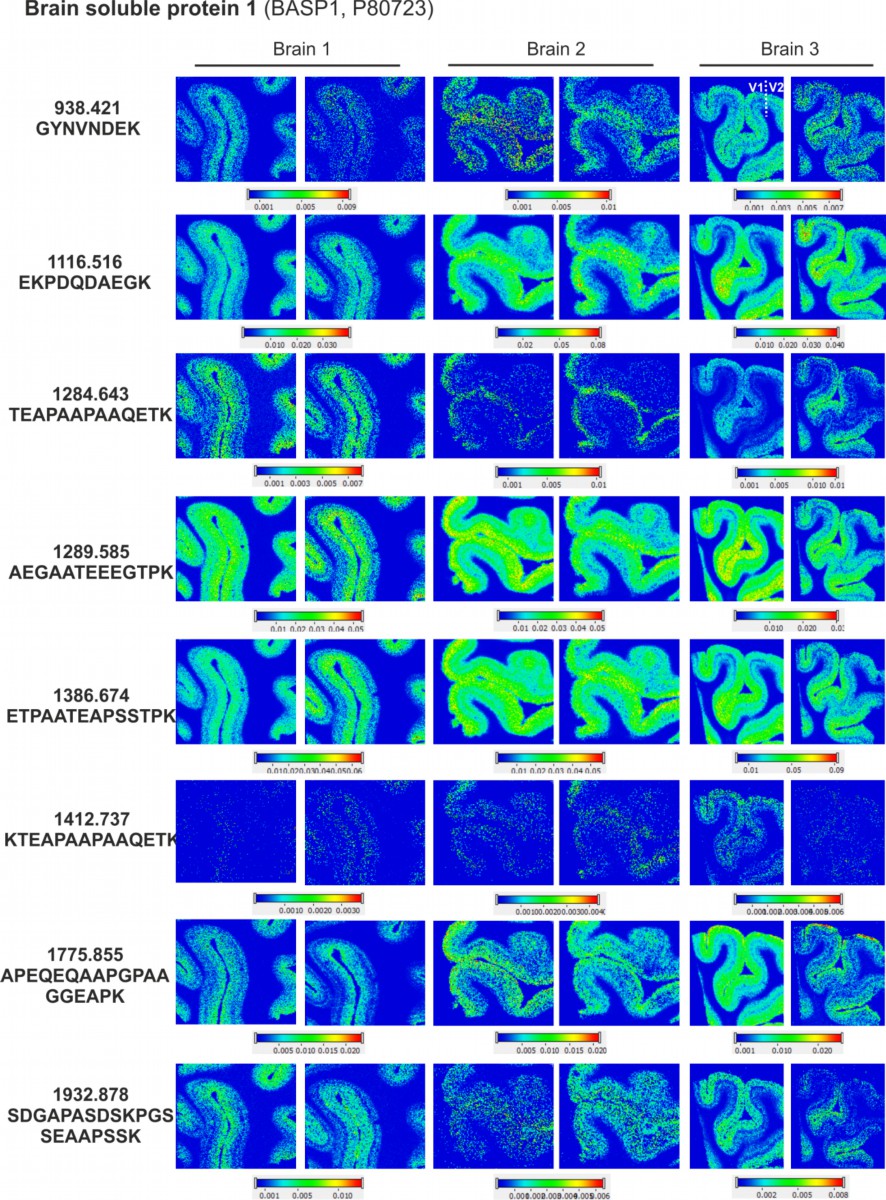


**Figure S2.** Anatomical distribution of tryptic peptides of brain soluble protein 1 in human V1, identified by MALDI-MS/MS after on tissue tryptic digest. The border between V1 and V2 is visualized in brain3. Two technical replicates were performed for each of the three brain specimen.


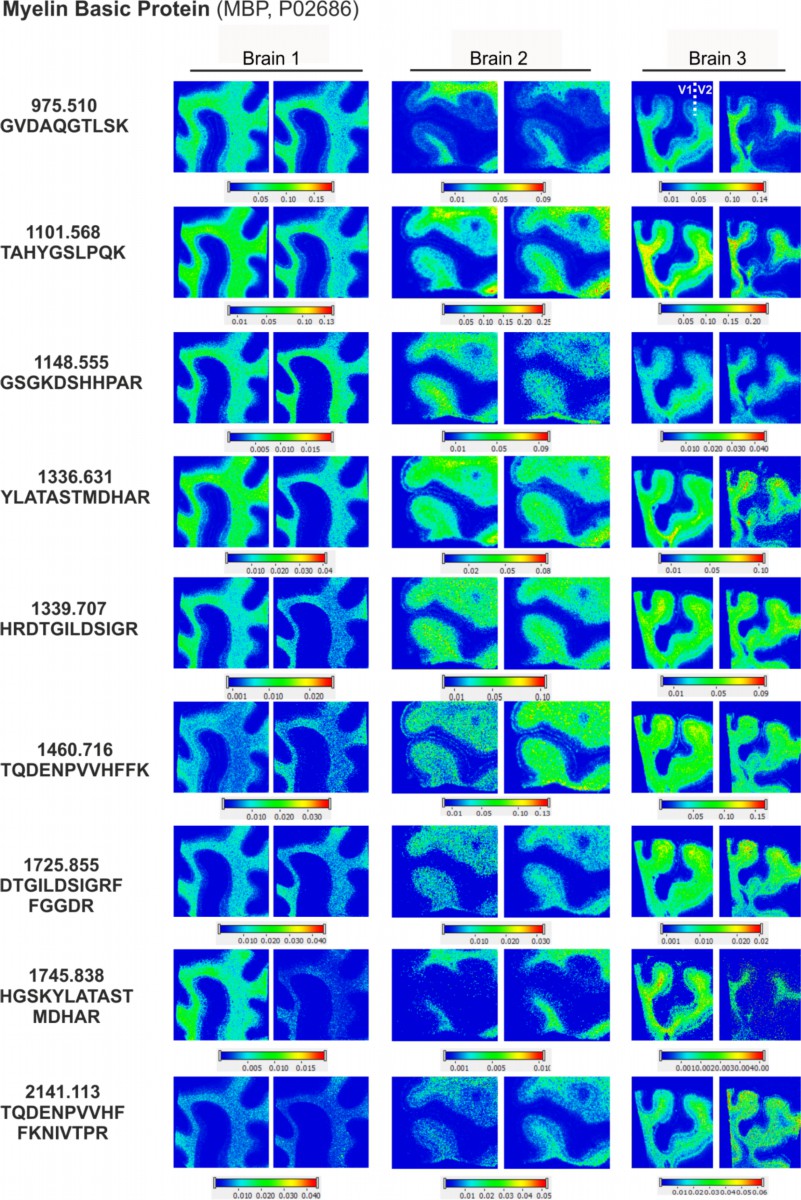


**Figure S3.** Anatomical distribution of tryptic peptides of myelin basic protein in human V1, identified by MALDI-MS/MS after on tissue tryptic digest. Layer IV defines the border V1 and V2. Two technical replicates were performed for each of the three brain specimen.


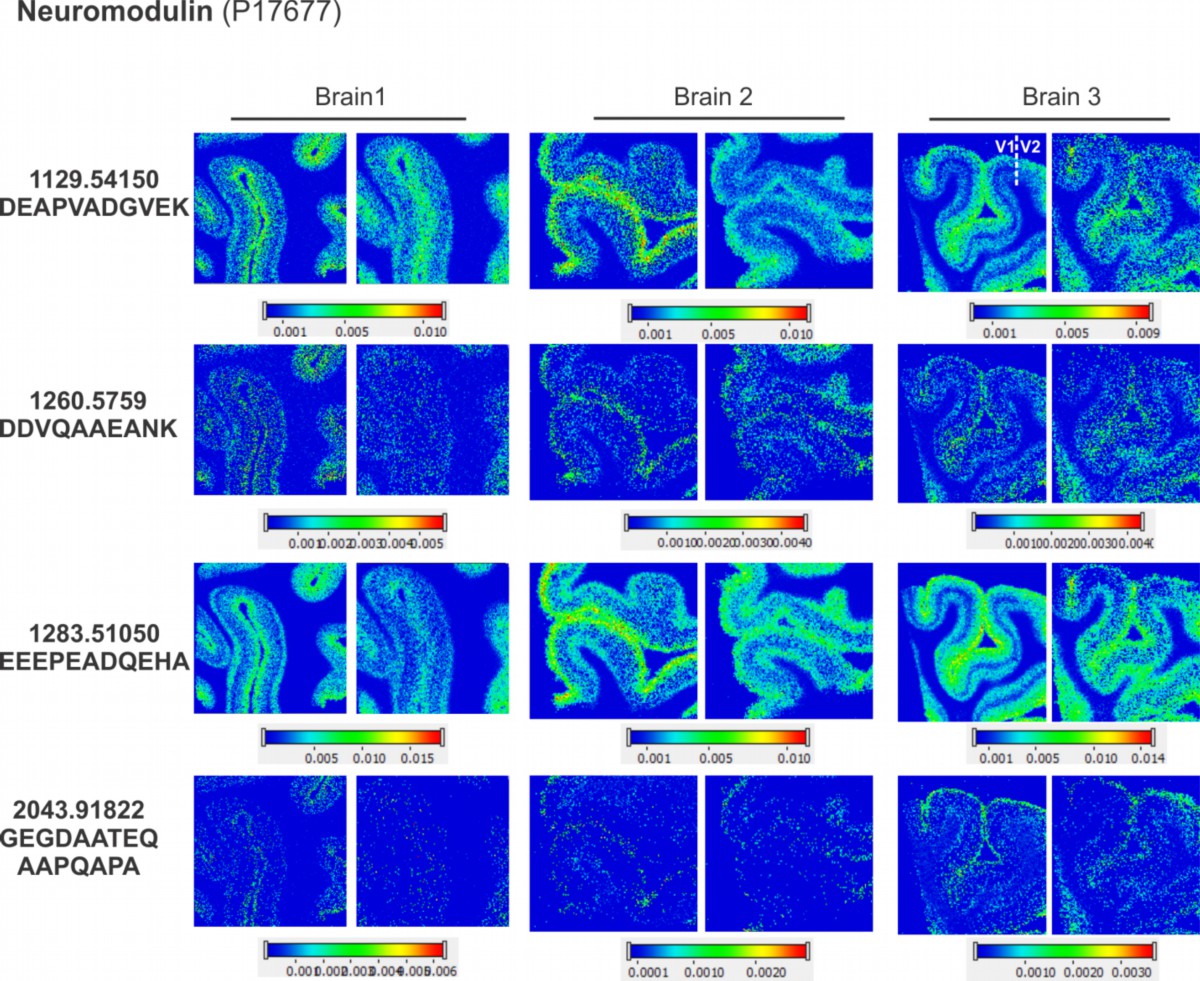


**Figure S4**. Anatomical distribution of tryptic peptides of neuromodulin (GAP43) in human V1, identified by MALDI-MS/MS after on tissue tryptic digest. The absence in layer IV defines the border V1 and V2.. Two technical replicates were performed for each of the three brain specimen.


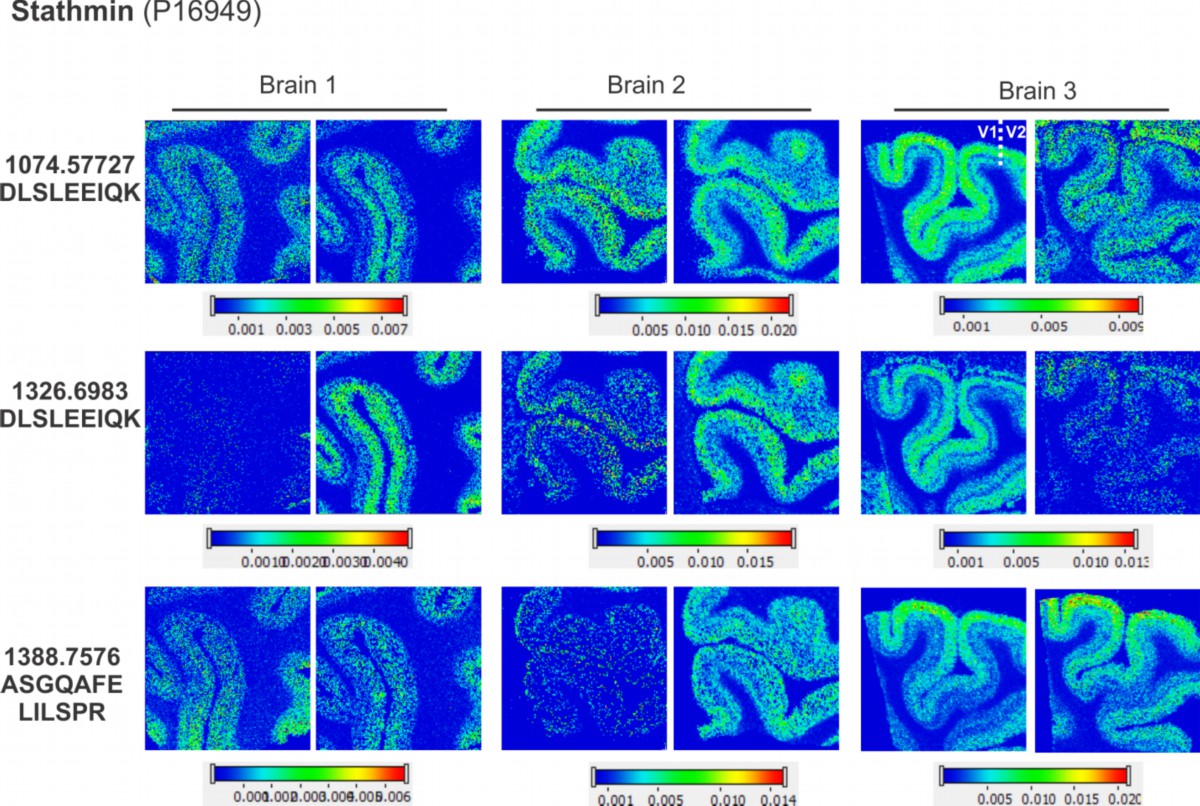


**Figure S5.** Anatomical distribution of tryptic peptides of stathmin in human V1, identified by MALDI-MS/MS after on tissue tryptic digest. A distinct absence in layer IV defines the border V1 and V2. Two technical replicates were performed for each of the three brain specimen.

**
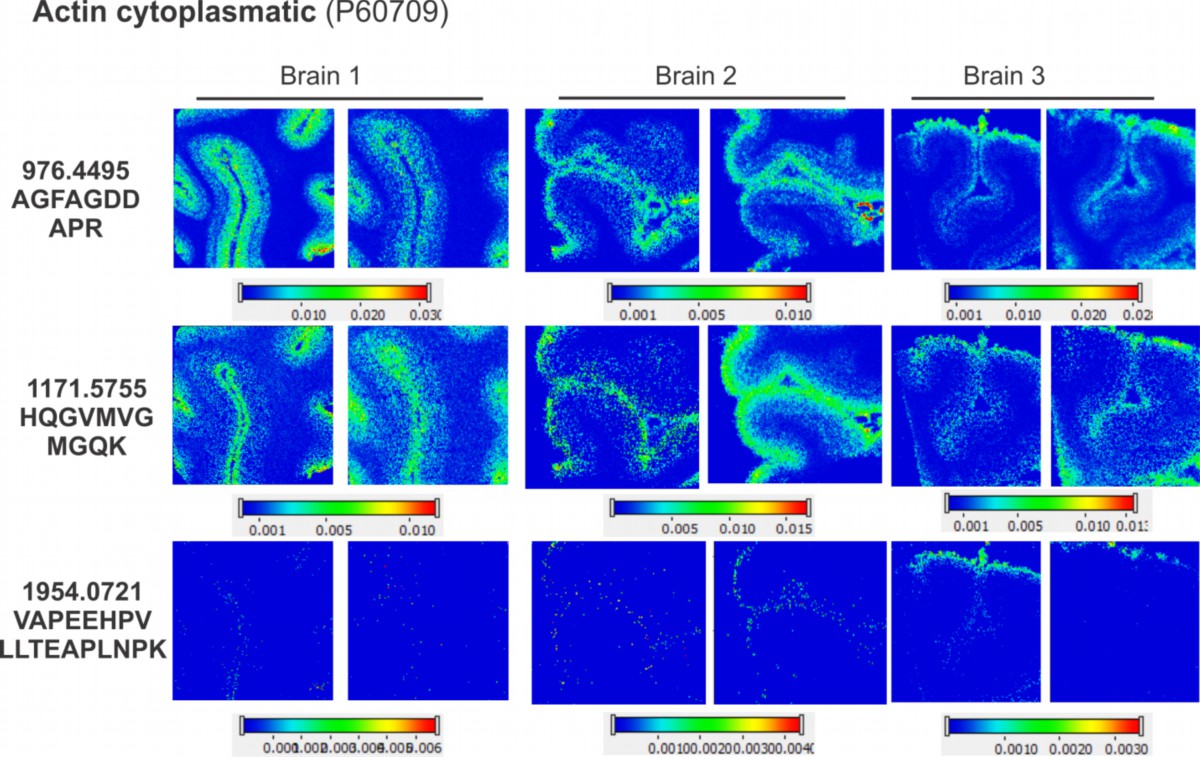
**

**Figure S8.** Anatomical distribution of tryptic peptides of cytoplasmic actin in human V1, identified by MALDI-MS/MS after on tissue tryptic digest. Two technical replicates were performed for each of the three brain specimen.


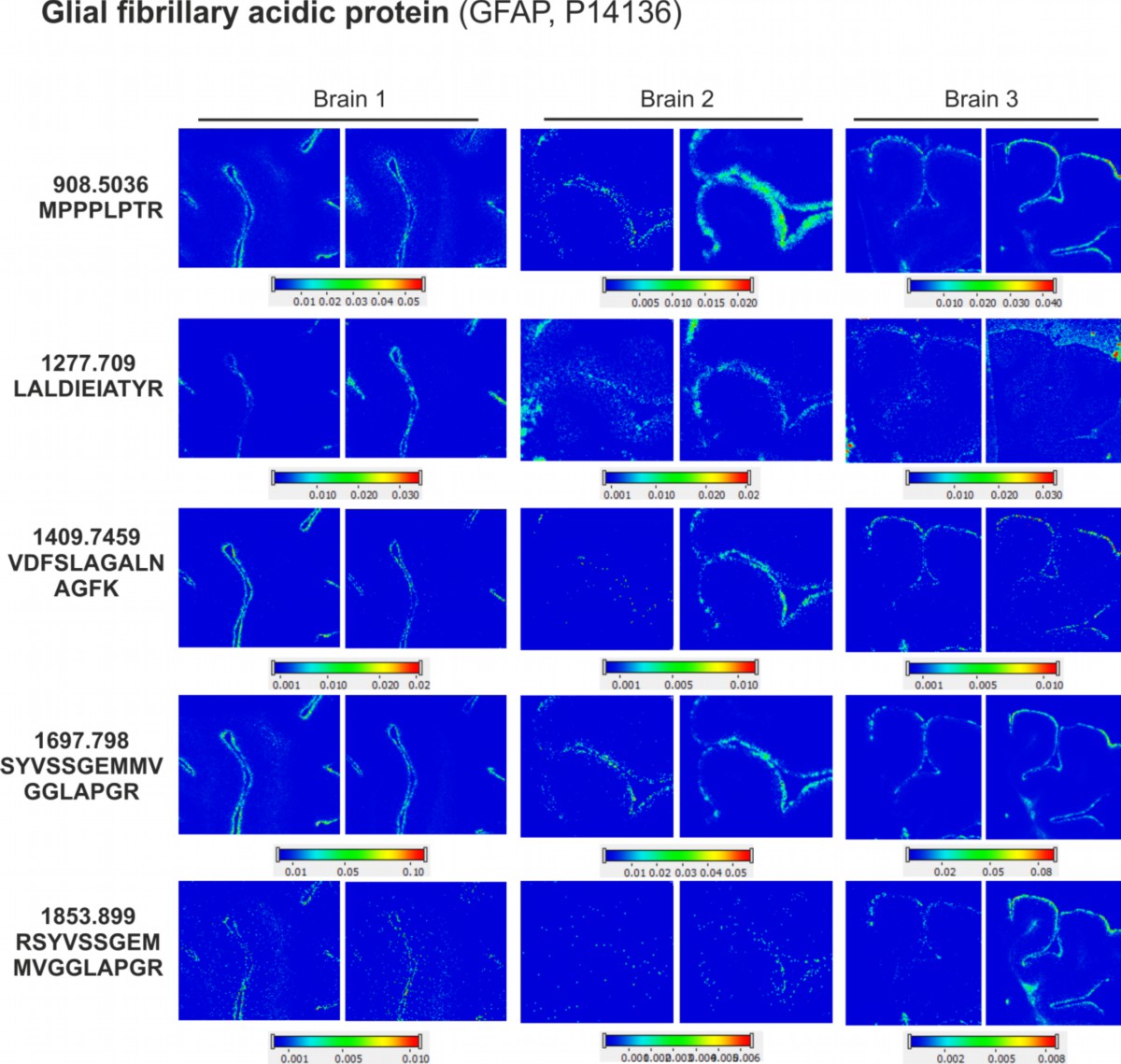


**Figure S7**. Anatomical distribution of tryptic peptides of glial fibrillary acidic protein (GFAP) in human V1, identified by MALDI-MS/MS after on tissue tryptic digest. Two technical replicates were performed for each of the three brain specimen.


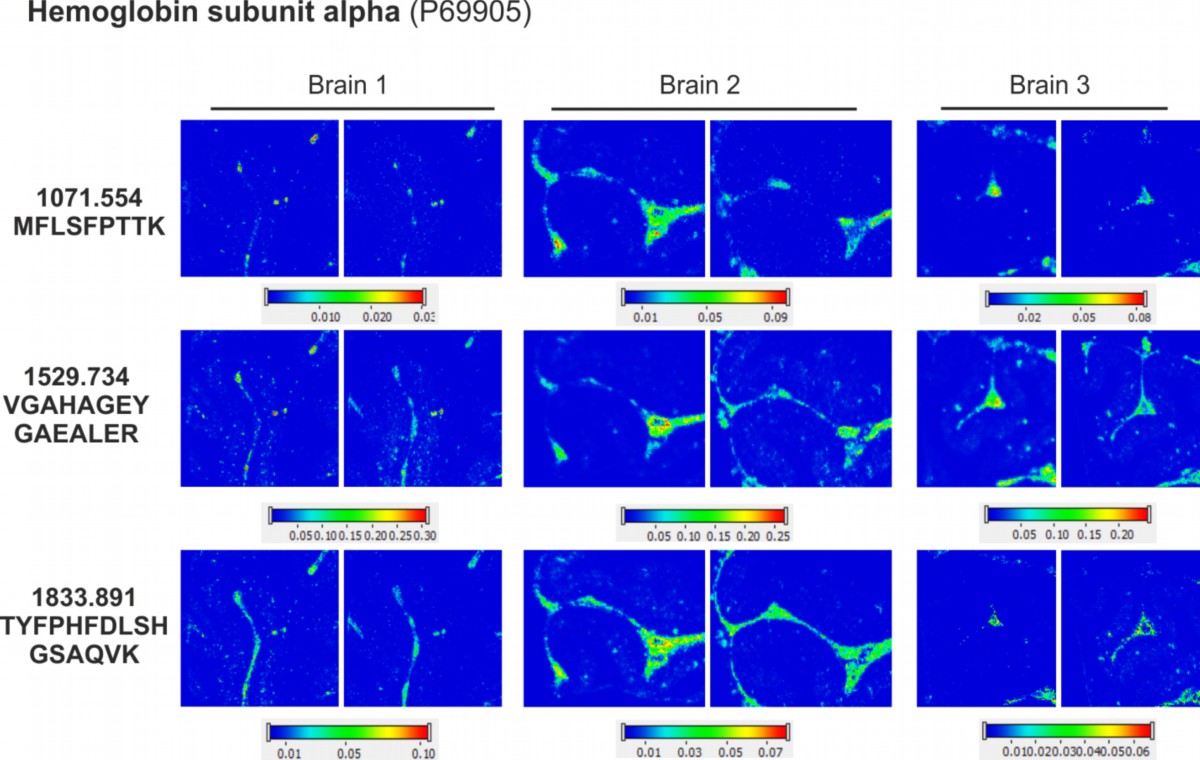


**Figure S8.** Anatomical distribution of tryptic peptides of hemoglobin α in human V1, identified by MALDI-MS/MS after on tissue tryptic digest. Two technical replicates were performed for each of the three brain specimen.


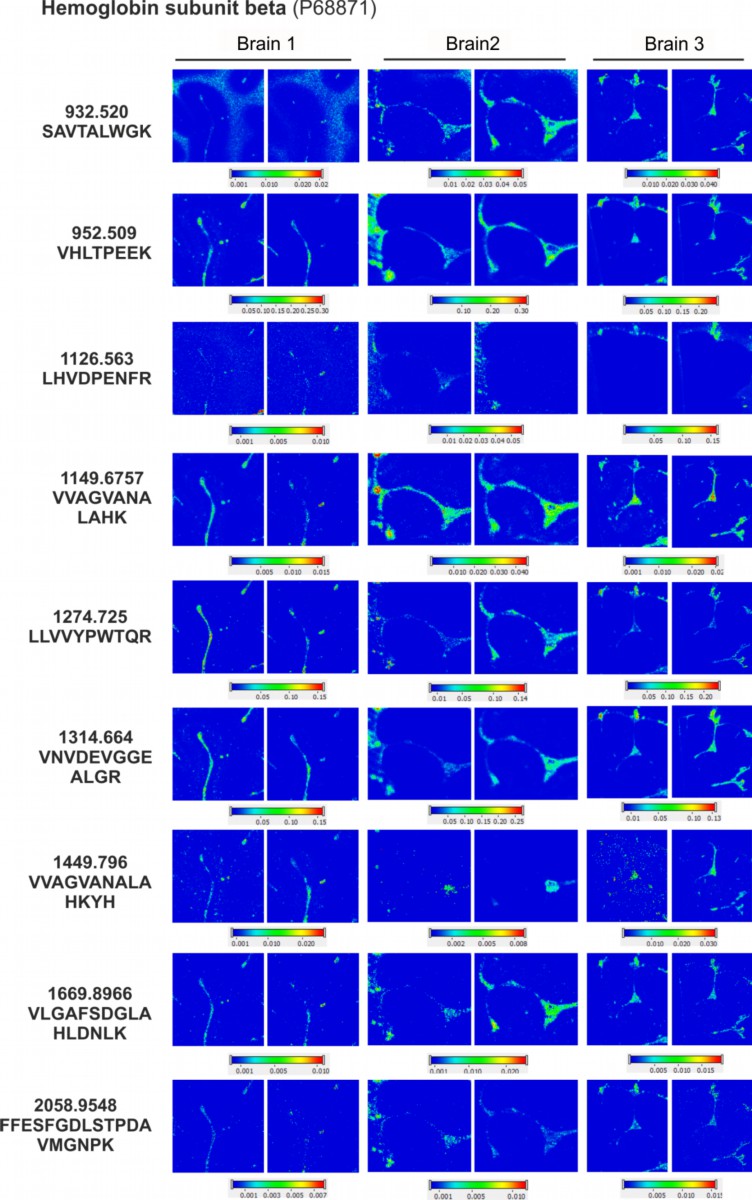


**Figure S9.** Anatomical distribution of tryptic peptides of hemoglobin ß in human V1, identified by MALDI-MS/MS after on tissue tryptic digest. Two technical replicates were performed for each of the three brain specimen.


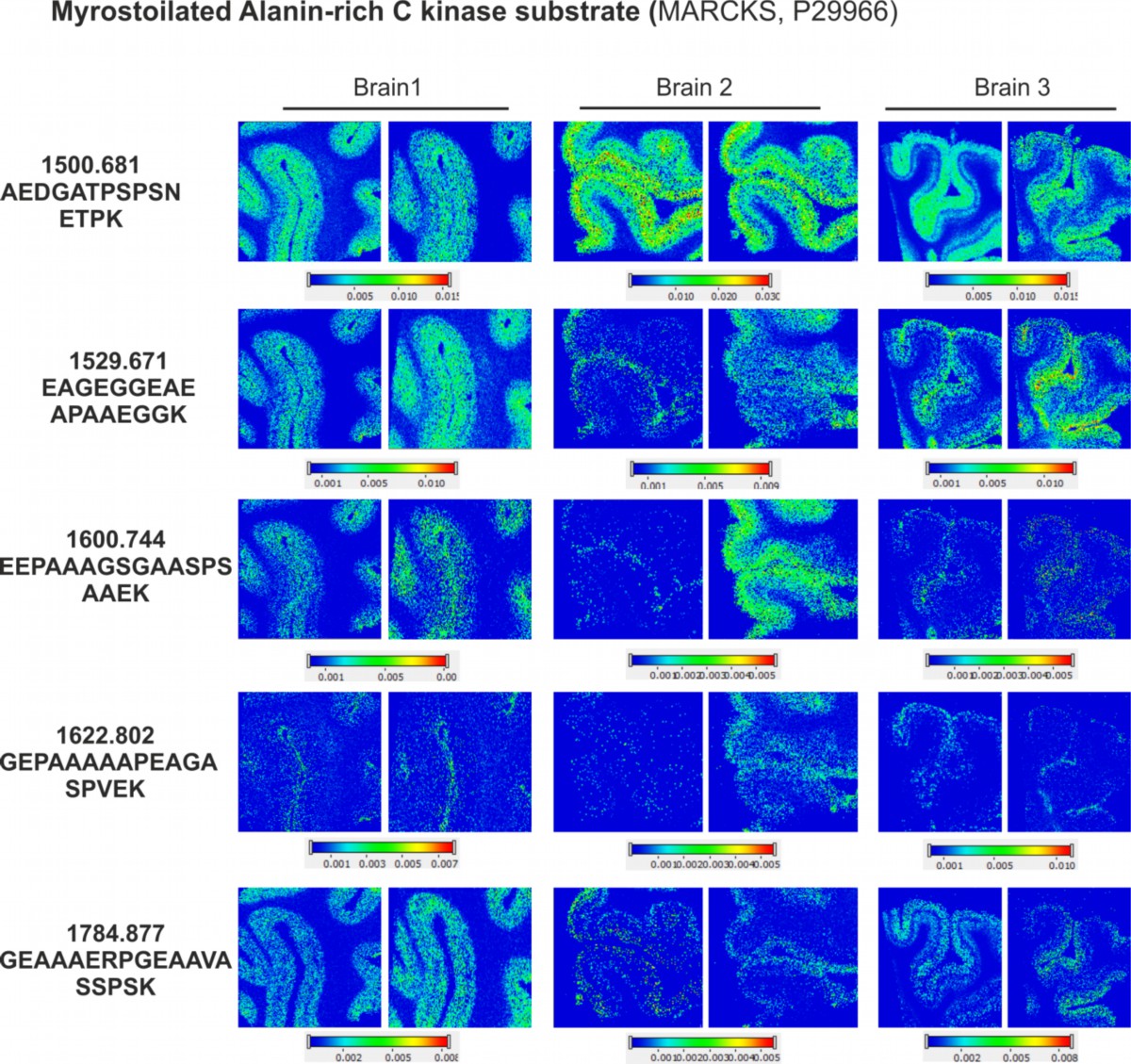


**Figure S10**. Anatomical distribution of tryptic peptides of myristoilated Alanin-rich C kinase substrate (MARCKS) in human V1, identified by MALDI-MS/MS after on tissue tryptic digest. Two technical replicates were performed for each of the three brain specimen.


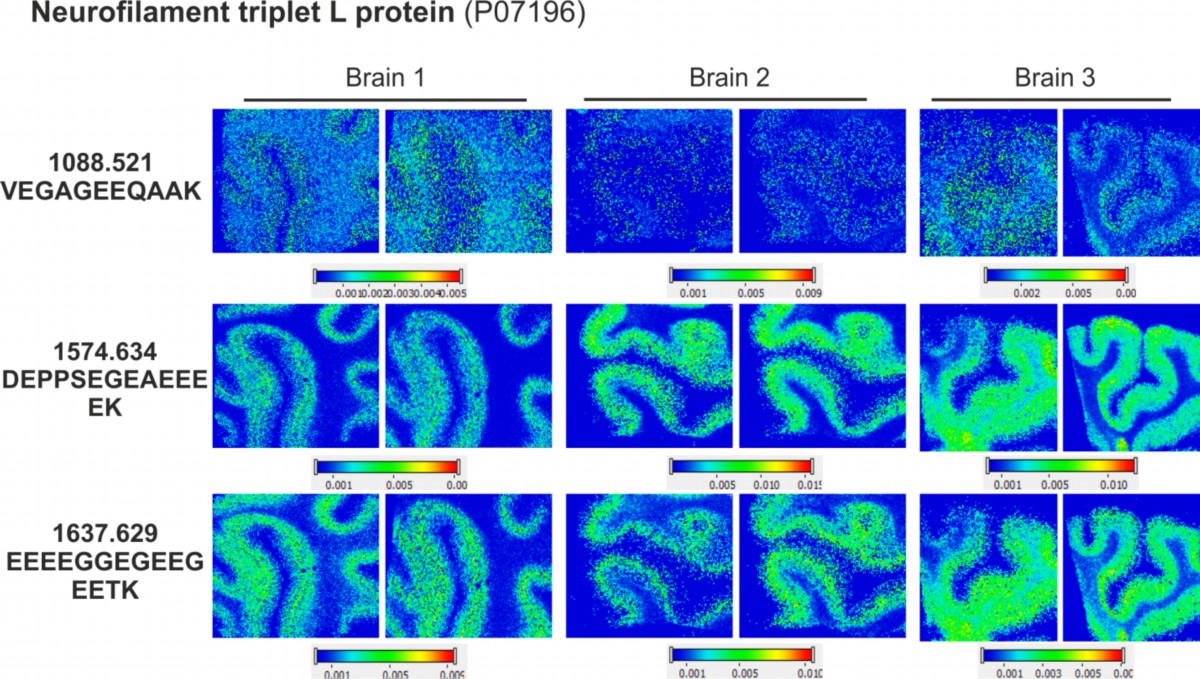


**Figure S11.** Anatomical distribution of tryptic peptides of neurofilament triplet L protein in human V1, identified by MALDI-MS/MS after on tissue tryptic digest. Two technical replicates were performed for each of the three brain specimen.


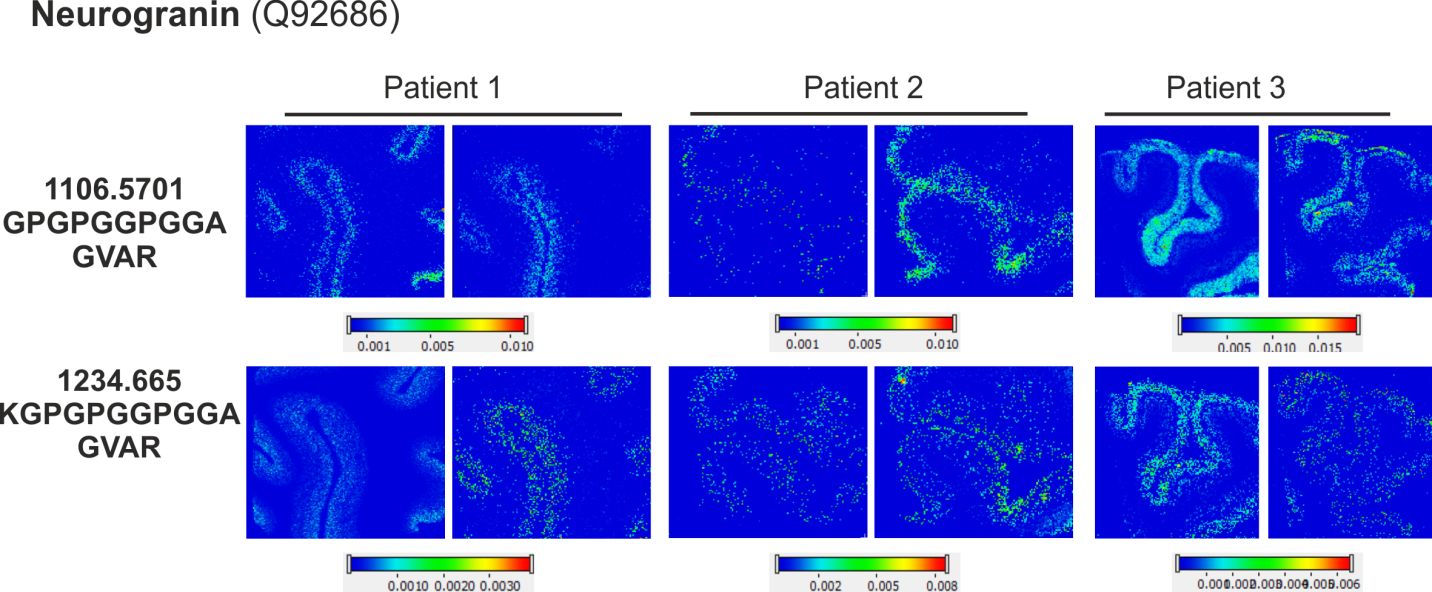


**Figure S12**. Anatomical distribution of tryptic peptides of neurogranin in human V1, identified by MALDI-MS/MS after on tissue tryptic digest. Two technical replicates were performed for each of the three brain specimen.


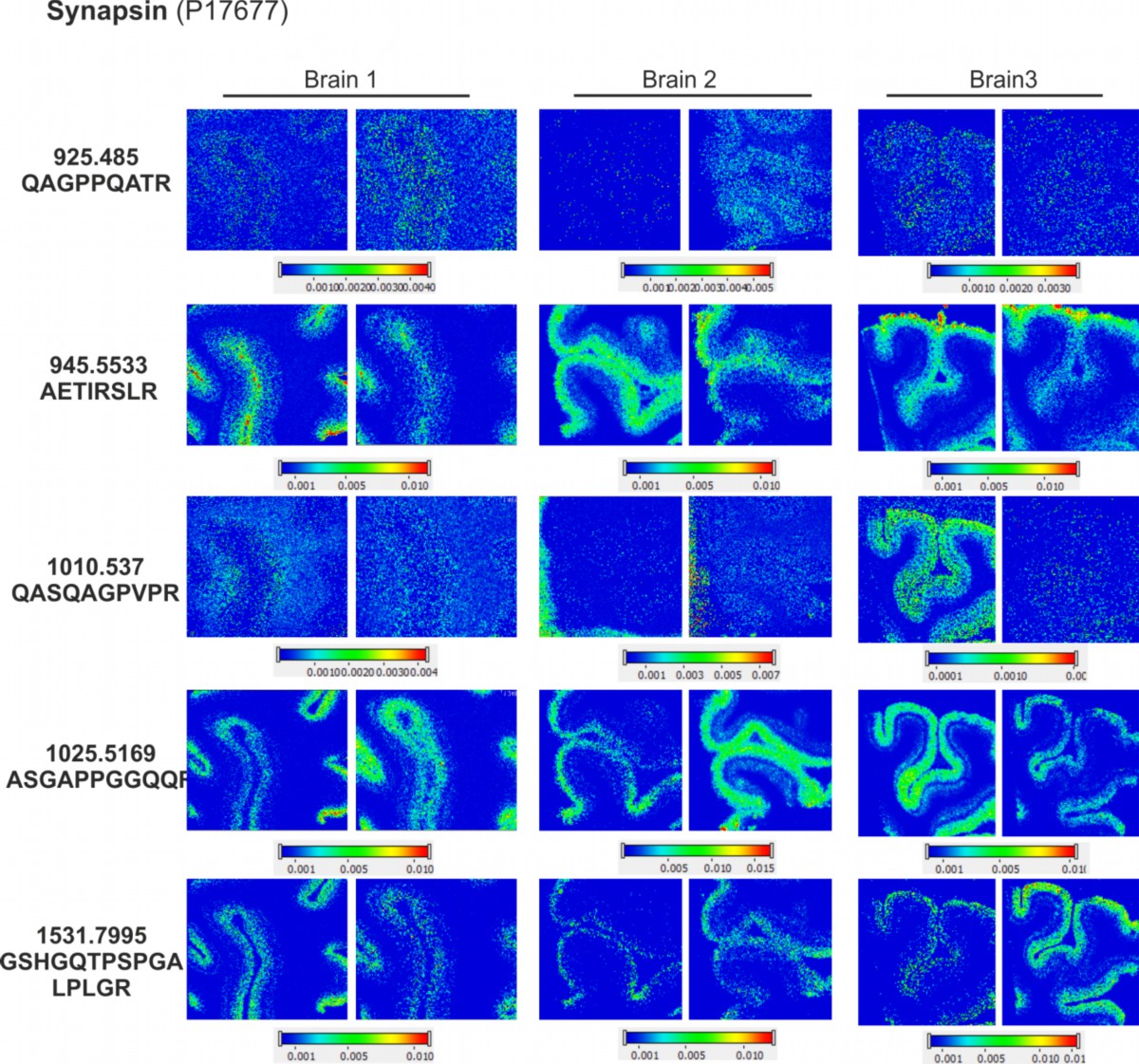
 **Figure S13.** Anatomical distribution of tryptic peptides of synapsin in human V1, identified by MALDI-MS/MS after on tissue tryptic digest. Two technical replicates were performed for each of the three brain specimen.


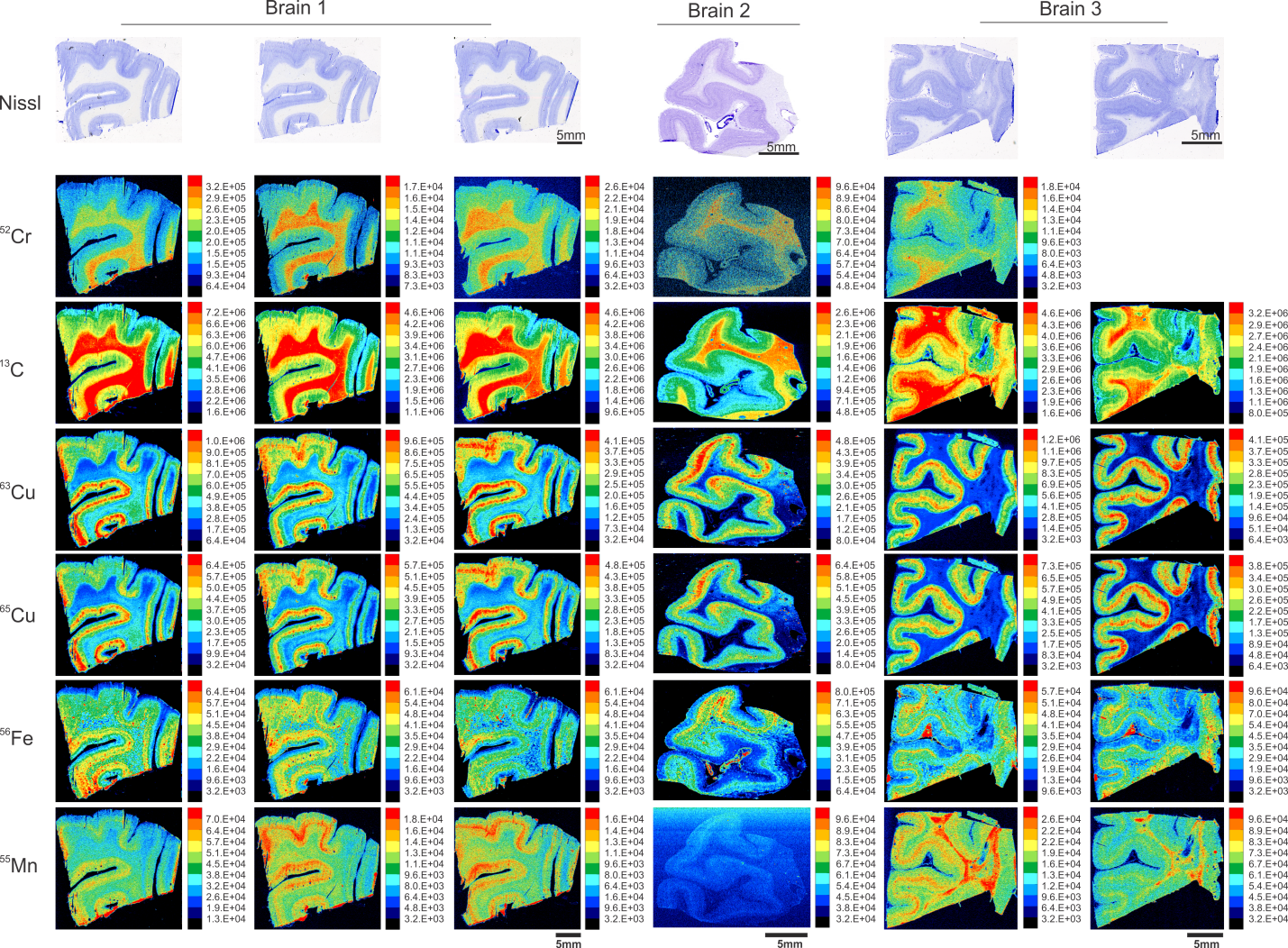


**Figure S14.** LA-ICP-MS images of the distribution of Cr, C, Cu, Fe and Mn in the primary visual cortex of three human brain samples. Scale bar: 5 mm, applies to all images of the same brain. Color scales represent element intensity in arbitrary units, individually scaled for each image.

**Table S2.** Lipid species visualized by MALDI-MSI, identified based on high resolution mass measurement in comparison with the theoretical m/z value. Cer, ceramide; DG; diglyceride; GlcCer, glucosylceramide; LPC, lysophosphatidylcholine; PC, phosphatidylcholine; PA, phosphatidic acid; PE, phosphatidylethanolamine; PS, phosphatidylserine; SM, sphingomyelin; PI-Cer, ceramide phosphoinositol.

| **m/z exp** |  | | **Lipid** | |  | |  | | **m/z teor** | |  | | **error(ppm)** | |
| --- | --- | --- | --- | --- | --- | --- | --- | --- | --- | --- | --- | --- | --- | --- |
|  |  |  | |  | |  | |  | |  | |  | |  |
| 504.3445 |  | [LPC_O-16:0 + Na] | | | |  | | 504.3424 | |  | | -4.16 | |  |
| 534.2949 |  | [LPC_16:0 + K]+ | | | |  | | 534.2956 | |  | | 1.31 | |  |
| 551.5027 |  | [DG_P-32:1 + H]+ | | | |  | | 551.5034 | |  | | 1.27 | |  |
| 573.4850 |  | [DG_P-32:1 + Na]+ | | | |  | | 573.4853 | |  | | 0.52 | |  |
| 577.5186 |  | [DG_O-32:0 + Na]+ | | | |  | | 577.5171 | |  | | -2.60 | |  |
| 599.5020 |  | [DG_P-34:2 + Na]+/[DG_O-34:3 + Na]+ | | | | | | 599.5015 | |  | | -0.83 | |  |
| 601.5193 |  | [DG_P-34:1+ Na]+/[DG_O-34:2 + Na]+ | | | | | | 601.5171 | |  | | -3.66 | |  |
| 603.5339 |  | [DG_P-34:0+ Na]+/[DG_O-34:1 + Na]+ | | | | | | 603.5328 | |  | | -1.82 | |  |
| 605.5501 |  | [DG_O-34:0 + Na]+ | | | |  | | 605.5484 | |  | | -2.81 | |  |
| 621.4842 |  | [DG_33:0 + Na]+ | | | |  | | 621.4855 | |  | | 2.09 | |  |
| 645.4853 |  | [PA_O-33:2 +H]+/[PA_P-33:1 + H]+ | | | | | | 645.4854 | |  | | 0.15 | |  |
| 649.5165 |  | [PA_O-33:0 +H]+ | | | |  | | 649.5167 | |  | | 0.31 | |  |
| 630.617 |  | [Cer_m40:0 + Na] | | | |  | | 630.6159 | |  | | -1.74 | |  |
| 669.4458 |  | [PA_32:1 + Na]+ | | | |  | | 669.4466 | |  | | 1.20 | |  |
| 685.4208 |  | [PA_32:1 + K]+ | | | |  | | 685.4205 | |  | | -0.44 | |  |
| 695.4622 |  | [PA_34:2 + Na] + | | | |  | | 695.4622 | |  | | 0.00 | |  |
| 697.4775 |  | [PA_34:0 + H]+ | | | |  | | 697.4803 | |  | | 4.01 | |  |
| 709.5139 |  | [PA_O-36:2 + Na]+/PA_P-36:1 + Na]+ | | | | | | 709.5143 | |  | | 0.56 | |  |
| 711.4352 |  | [PA_34:2 + K]+ | | | |  | | 711.4362 | |  | | 1.41 | |  |
| 713.4524 |  | [PA_34:1 + K]+ | | | |  | | 713.4518 | |  | | -0.84 | |  |
| 718.5745 |  | [PC_O-32:1 + H]+ | | | |  | | 718.5745 | |  | | 0.01 | |  |
| 723.4937 |  | [PA_36:2 + Na]+ | | | |  | | 723.4935 | |  | | -0.28 | |  |
| 725.5574 |  | [SM_d34:1 +Na]+ | | | |  | | 725.5568 | |  | | -0.83 | |  |
| 727.5111 |  | [SM_d33:1 + K]+ | | | |  | | 727.5151 | |  | | 5.50 | |  |
| 728.5205 |  | [PC_30:0 + Na]+ | | | |  | | 728.5201 | |  | | -0.55 | |  |
| 734.5704 |  | [PC_32:0 + H]+ | | | |  | | 734.5694 | |  | | -1.36 | |  |
| 737.4511 |  | [PA_36:3 + K]+ | | | |  | | 737.4518 | |  | | 0.95 | |  |
| 739.4676 |  | [PA_36:2 + K]+ | | | |  | | 739.4675 | |  | | -0.14 | |  |
| 741.5313 |  | [SM_d34:1 +K]+ | | | |  | | 741.5307 | |  | | -0.81 | |  |
| 742.5337 |  | [PE_36:3+H]+ | | | |  | | 742.5340 | |  | | 0.40 | |  |
| 744.4931 |  | [PC_30:0 + K]+ | | | |  | | 744.4940 | |  | | 1.21 | |  |
| 744.5542 |  | [PE_36:2+H]+ | | | |  | | 744.5538 | |  | | -0.54 | |  |
| 744.5907 |  | [PC_O-34:2/P-34:1+H]+ | | | | | | 744.5902 | |  | | -0.67 | |  |
| 746.6062 |  | [PC_O-16:0/18:1+H]+ | | | | | | 746.6058 | |  | | -0.54 | |  |
| 748.5850 |  | [PC_33:0 + H]+ | | | |  | | 748.5851 | |  | | 0.13 | |  |
| 751.5248 |  | [PA_38:2 + Na]+ | | | |  | | 751.5248 | |  | | 0.00 | |  |
| 751.5719 |  | [PA_38:2 + Na]+ | | | |  | | 751.5724 | |  | | 0.67 | |  |
| 753.5282 |  | [PE-Cer_d38:2 + K]+ | | | |  | | 753.5307 | |  | | 3.32 | |  |
| 753.5875 |  | [SM_d36:1 + Na]+ | | | |  | | 753.5881 | |  | | 0.80 | |  |
| 754.5355 |  | [PC_32:1 +Na]+ | | | |  | | 754.5357 | |  | | 0.27 | |  |
| 756.5510 |  | [PC_32:0 +Na]+ | | | |  | | 756.5514 | |  | | 0.53 | |  |
| 758.5093 |  | [PC_31:0 + K]+ | | | |  | | 758.5097 | |  | | 0.53 | |  |
| 759.6373 |  | [SM_d38:1 + H]+ | | | |  | | 759.6374 | |  | | 0.13 | |  |
| 760.5855 |  | [PC_34:1 + H]+ | | | |  | | 760.5851 | |  | | -0.53 | |  |
| 761.4517 |  | [PA_38:5 + k]+ | | | |  | | 761.4518 | |  | | 0.13 | |  |
| 763.4668 |  | [PA_38:4+ k]+ | | | |  | | 763.4675 | |  | | 0.92 | |  |
| 765.4835 |  | [PA_38:3+ k]+ | | | |  | | 765.4831 | |  | | -0.52 | |  |
| 766.5594 |  | [GlcCer_36:1 + K] | | | |  | | 766.5594 | |  | | 0.00 | |  |
| 767.4980 |  | [PA_38:2+ k]+ | | | |  | | 767.4988 | |  | | 1.04 | |  |
| 767.5441 |  | [SM_36:2 + K]+ | | | |  | | 767.5464 | |  | | 2.94 | |  |
| 768.5516 |  | [PC_35:4 + H]+ | | | |  | | 768.5538 | |  | | 2.86 | |  |
| 768.5879 |  | [PC_O-34:1 + H]+ | | | |  | | 768.5902 | |  | | 2.99 | |  |
| 769.4762 |  | [PA_40:7 + Na] + | | | |  | | 769.4779 | |  | | 2.21 | |  |
| 769.5611 |  | [SM_d36:1 + K]+ | | | |  | | 769.5620 | |  | | 1.17 | |  |
| 770.5098 |  | [PC_32:1 + K]+ | | | |  | | 770.5097 | |  | | -0.13 | |  |
| 770.5644 |  | [PC_33:0 + Na]+ | | | |  | | 770.5670 | |  | | 3.37 | |  |
| 772.526 |  | [PC_32:0 + K] + | | | |  | | 772.5253 | |  | | -0.91 | |  |
| 774.6011 |  | [PC_35:1 + H] +/[PE_38:1 + H]+ | | | | | | 774.6007 | |  | | -0.52 | |  |
| 776.5906 |  | [HexCer_d38:2 +Na]+ | | | | | | 776.5928 | |  | | 2.83 | |  |
| 778.6082 |  | [CerP_d44:2 + Na]+ | | | |  | | 778.6085 | |  | | 0.39 | |  |
| 780.5512 |  | [PC_34:2 + Na] + | | | |  | | 780.5514 | |  | | 0.26 | |  |
| 781.6194 |  | [SM_d38:1 + Na]+ | | | |  | | 781.6194 | |  | | 0.00 | |  |
| 782.5087 |  | [PC_33:2 + K] + | | | |  | | 782.5097 | |  | | 1.28 | |  |
| 782.5677 |  | [PC_34:1 + Na] + | | | |  | | 782.5670 | |  | | -0.89 | |  |
| 784.5245 |  | [PC_33:1 + K] + | | | |  | | 784.5253 | |  | | 1.02 | |  |
| 784.5796 |  | [PC_34:0 + Na] + | | | |  | | 784.5827 | |  | | 3.95 | |  |
| 785.4502 |  | [PA_40:7 + K] + | | | |  | | 785.4518 | |  | | 2.04 | |  |
| 785.6518 |  | [SM_d40:2 + H]+ | | | |  | | 785.6531 | |  | | 1.65 | |  |
| 786.5993 |  | [PC_36:2 + H] + | | | |  | | 786.6007 | |  | | 1.78 | |  |
| 788.6163 |  | [PC_36:1 + H] + | | | |  | | 788.6162 | |  | | -0.13 | |  |
| 790.5139 |  | [PE_O-38:5 + K]+/[PE_P-38:4 + Na] | | | | | | 790.5147 | |  | | 1.01 | |  |
| 792.5672 |  | [HexCer_d38:2 + K]+ | | | |  | | 792.5755 | |  | | 10.47 | |  |
| 794.5812 |  | [CerP_d44:2 + K]+ | | | |  | | 794.5824 | |  | | 1.51 | |  |
| 794.6022 |  | [PC_O-38:5/P-38:4] | | | |  | | 794.6058 | |  | | 4.53 | |  |
| 796.5246 |  | [PC_34:2 + K] + | | | |  | | 796.5253 | |  | | 0.88 | |  |
| 796.5807 |  | [PC_35:2 + Na] + | | | |  | | 796.5827 | |  | | 2.51 | |  |
| 797.5926 |  | [SM_d38:1 + K]+ | | | |  | | 797.5933 | |  | | 0.88 | |  |
| 798.5412 |  | [PC_34:1 + K]+ | | | |  | | 798.5410 | |  | | -0.25 | |  |
| 798.5945 |  | [PC_35:0 + Na]+ | | | |  | | 798.5983 | |  | | 4.76 | |  |
| 799.6663 |  | [SM_d41:2 + H]+ | | | |  | | 799.6687 | |  | | 3.00 | |  |
| 804.5504 |  | [PC_36:4 + Na]+ | | | |  | | 804.5514 | |  | | 1.24 | |  |
| 806.5676 |  | [PC_36:3 + Na]+ | | | |  | | 806.5670 | |  | | -0.74 | |  |
| 806.5828 |  | 1-(10-methylhexadecanyl)-2-(8-[3]-ladderane-octanyl)-sn-glycerophosphocholine | | | | | | 806.5824 | |  | | -0.50 | |  |
| 808.5826 |  | [PC_36:2 + Na]+ | | | |  | | 808.5827 | |  | | 0.12 | |  |
| 808.6664 |  | [GlcCer_d40:0 + Na]+/[GalCer_d40:0 + Na] | | | | | | 808.6637 | |  | | -3.34 | |  |
| 809.6485 |  | [SM_d40:1 + Na]+ | | | |  | | 809.6507 | |  | | 2.72 | |  |
| 810.5988 |  | [PC_36:1 + Na]+ | | | |  | | 810.5983 | |  | | -0.62 | |  |
| 813.6835 |  | [SM_d42:2 + H]+ | | | |  | | 813.6844 | |  | | 1.11 | |  |
| 815.6978 |  | [SM_d42:1 + H]+ | | | |  | | 815.7000 | |  | | 2.70 | |  |
| 816.6472 |  | [PC_38:1 + H]+ | | | |  | | 816.6477 | |  | | 0.61 | |  |
| 820.5255 |  | [PC_36:4 + K]+ | | | |  | | 820.5253 | |  | | -0.24 | |  |
| 822.5406 |  | [PC_36:3 + K]+ | | | |  | | 822.541 | |  | | 0.49 | |  |
| 824.5568 |  | [PC_36:2 + K]+ | | | |  | | 824.5566 | |  | | -0.24 | |  |
| 825.6247 |  | [SM_d40:1 + K]+ | | | |  | | 825.6246 | |  | | -0.12 | |  |
| 826.5714 |  | [PC_36:1 + K]+ | | | |  | | 826.5723 | |  | | 1.09 | |  |
| 828.5142 |  | [PS_36:1 + K] | | | |  | | 828.5142 | |  | | 0.00 | |  |
| 828.5503 |  | [PC_38:6 + Na]+ | | | |  | | 828.5514 | |  | | 1.30 | |  |
| 830.5090 |  | [PE_40:6 + K]+ | | | |  | | 830.5097 | |  | | 0.84 | |  |
| 830.5660 |  | [PC_38:5 + Na]+ | | | |  | | 830.567 | |  | | 1.20 | |  |
| 832.5819 |  | [PC_38:4 + Na]+ | | | |  | | 832.5827 | |  | | 0.96 | |  |
| 832.6636 |  | [GlcCer_d42:2 + Na]+ | | | | | | 832.6637 | |  | | 0.12 | |  |
| 834.6782 |  | [GlcCer_d42:1 + Na]+ | | | | | | 834.6793 | |  | | 1.32 | |  |
| 835.6654 |  | [SM_d42:2 +Na]+ | | | |  | | 835.6663 | |  | | 1.08 | |  |
| 836.5152 |  | [PC_36:4 + K]+ | | | |  | | 836.5202 | |  | | 5.98 | |  |
| 836.6133 |  | [PC_38:2 + Na]+ | | | |  | | 836.6140 | |  | | 0.84 | |  |
| 837.6246 |  | [SM_d41:2 +K]+ | | | |  | | 837.6246 | |  | | 0.00 | |  |
| 837.6796 |  | [SM_d40:1 +Na]+ | | | |  | | 837.6820 | |  | | 2.87 | |  |
| 838.6170 |  | [PICer_d38:0 + H] | | | |  | | 838.6168 | |  | | -0.24 | |  |
| 839.6389 |  | [SM_d41:1 +K]+ | | | |  | | 839.6403 | |  | | 1.67 | |  |
| 841.7157 |  | [SM_d44:2 +H]+ | | | |  | | 841.7157 | |  | | 0.00 | |  |
| 844.5252 |  | [PC_38:6 + K]+ | | | |  | | 844.5253 | |  | | 0.12 | |  |
| 846.5389 |  | [PC_38:5 + K]+ | | | |  | | 846.5410 | |  | | 2.48 | |  |
| 848.5553 |  | [PC_38:4 + K]+ | | | |  | | 848.5566 | |  | | 1.53 | |  |
| 848.6368 |  | [GlcCer_d42:2+ K]+ | | | |  | | 848.6375 | |  | | 0.82 | |  |
| 851.6387 |  | [SM_d42:2 + K]+ | | | |  | | 851.6403 | |  | | 1.88 | |  |
| 851.6762 |  | 20:3-Glc-Campesterol | | | | | | 851.6759 | |  | | -0.35 | |  |
| 851.6952 |  | [SM_d43:2 + Na]+ | | | |  | | 851.6976 | |  | | 2.82 | |  |
| 852.4913 |  | [PE_42:9 + K] | | | |  | | 852.4940 | |  | | 3.17 | |  |
| 852.6422 |  | [PE_39:1 + Na]+ | | | |  | | 852.6453 | |  | | 3.64 | |  |
| 853.6545 |  | [SM_d42:1 + K]+ | | | |  | | 853.6559 | |  | | 1.64 | |  |
| 856.5810 |  | [PC_40:6 + Na]+ | | | |  | | 856.5827 | |  | | 1.98 | |  |
| 862.6524 |  | [PS_41:0 + Na]+ | | | |  | | 862.6532 | |  | | 0.93 | |  |
| 864.6328 |  | [PI-Cer_d40:1 + H] | | | |  | | 864.6324 | |  | | -0.46 | |  |
| 865.6544 |  | [SM_d43:2 + K]+ | | | |  | | 865.6559 | |  | | 1.73 | |  |
| 866.6465 |  | [PI-Cer_d40:0 + H] | | | |  | | 866.6481 | |  | | 1.85 | |  |
| 870.5394 |  | [PC_40:7 + K]+ | | | |  | | 870.5410 | |  | | 1.84 | |  |
| 872.5567 |  | [PC_40:6 + K]+ | | | |  | | 872.5566 | |  | | -0.11 | |  |
| 874.4981 |  | [PS_40:6 + K]+ | | | |  | | 874.4995 | |  | | 1.60 | |  |
| 876.6693 |  | [GlcCer_d44:2 + K]+ | | | |  | | 876.6689 | |  | | -0.46 | |  |
| 879.6709 |  | [SM_d42:2 + K]+ | | | |  | | 879.6716 | |  | | 0.80 | |  |
| 896.4821 |  | [PS_42:9 + K]+ | | | |  | | 896.4838 | |  | | 1.90 | |  |
|  |  |  | |  | |  | |  | |  | |  | |  |
|  |  |  | |  | |  | |  | |  | |  | |  |
|  |  |  | |  | |  | |  | |  | |  | |  |


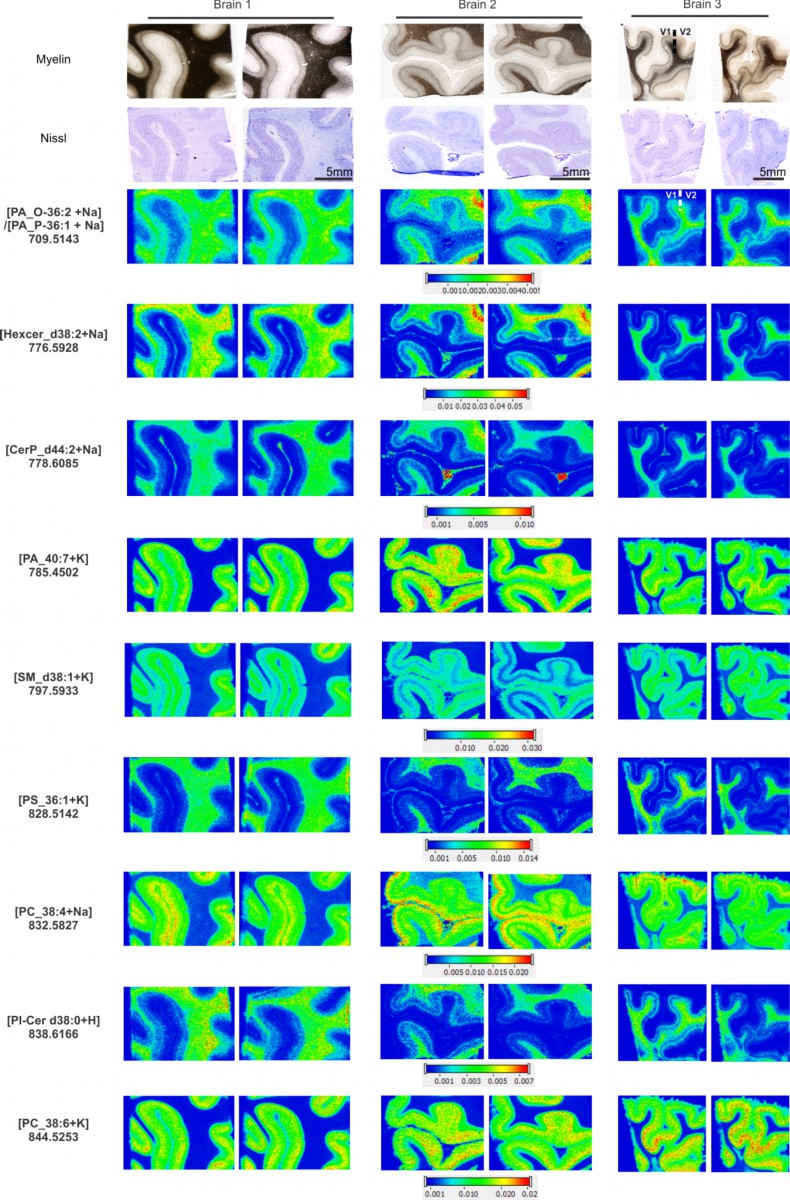


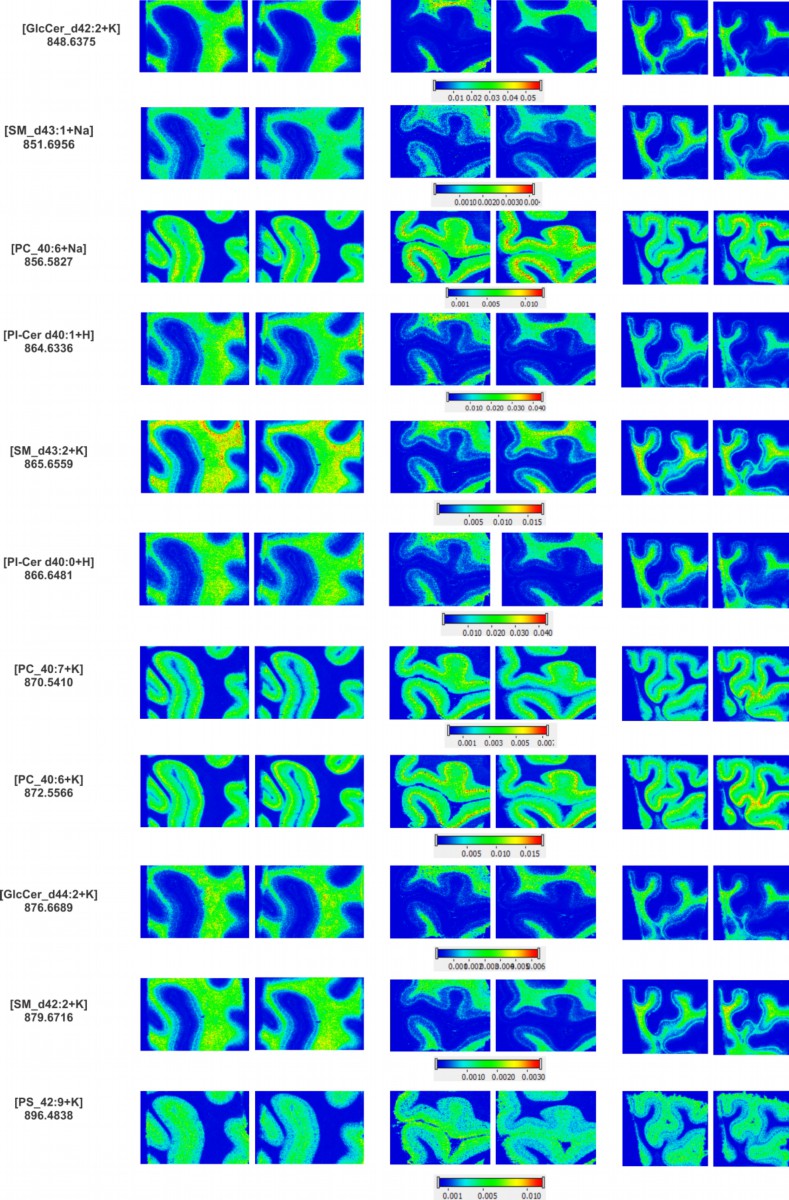


**Figure S15**. Anatomical distribution of lipid species with distinct laminar distribution pattern in the primary visual cortex (area V1) of three human brain samples.


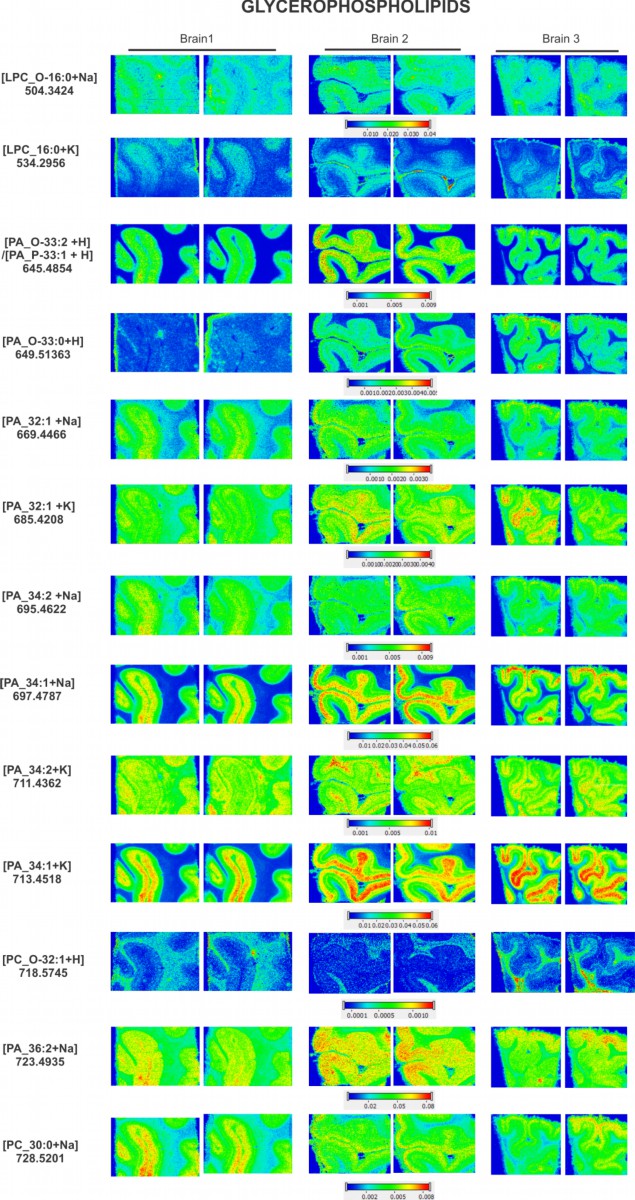


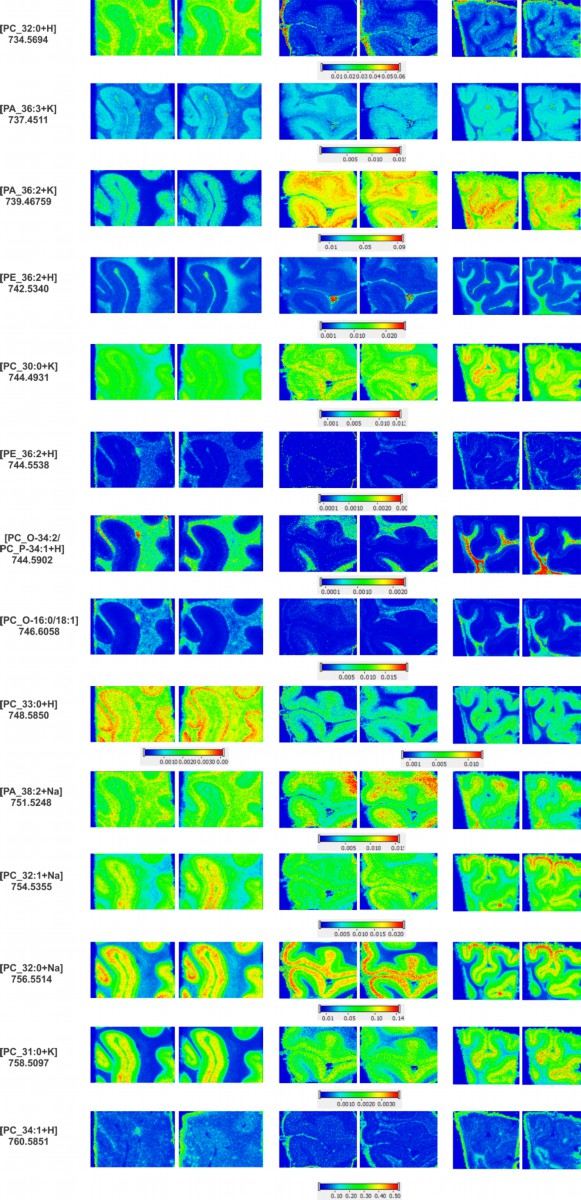

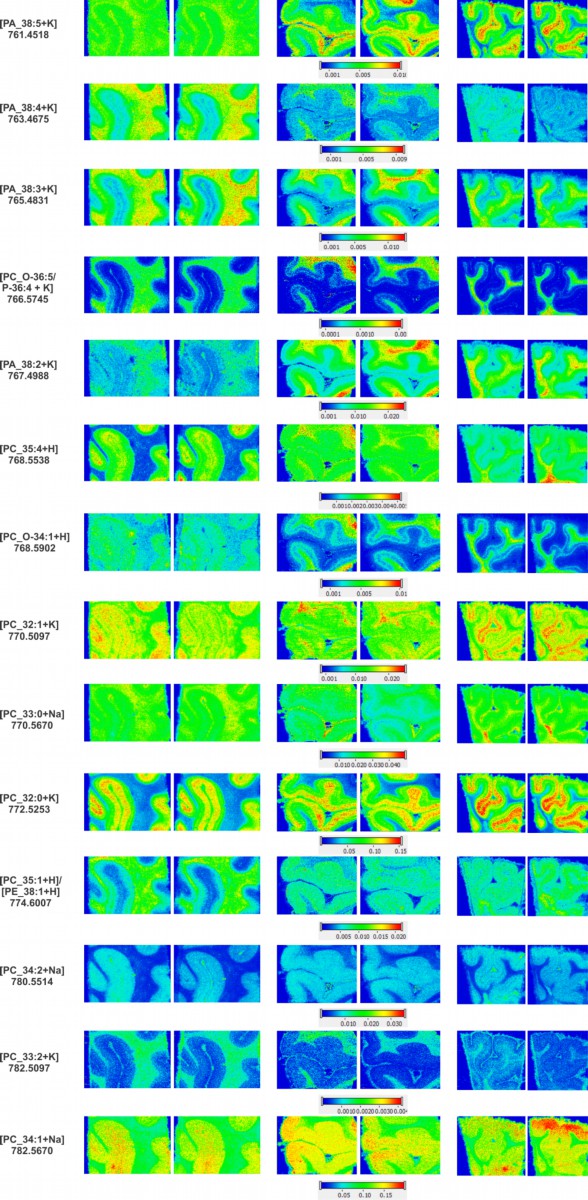


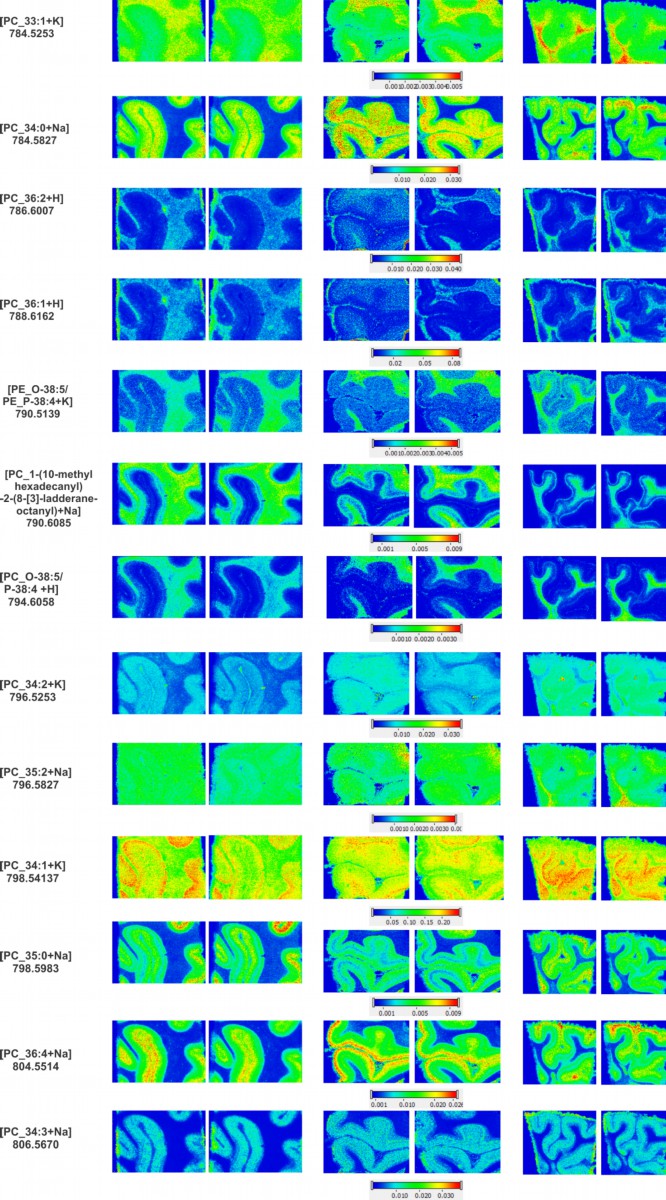

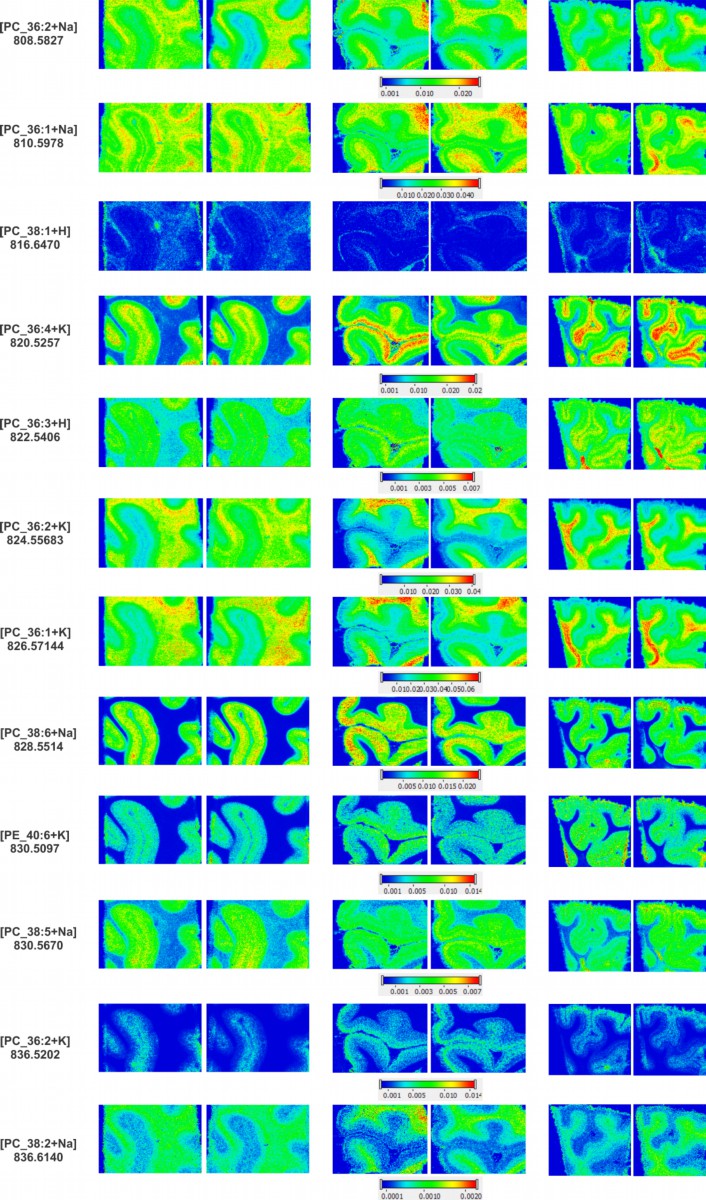
s
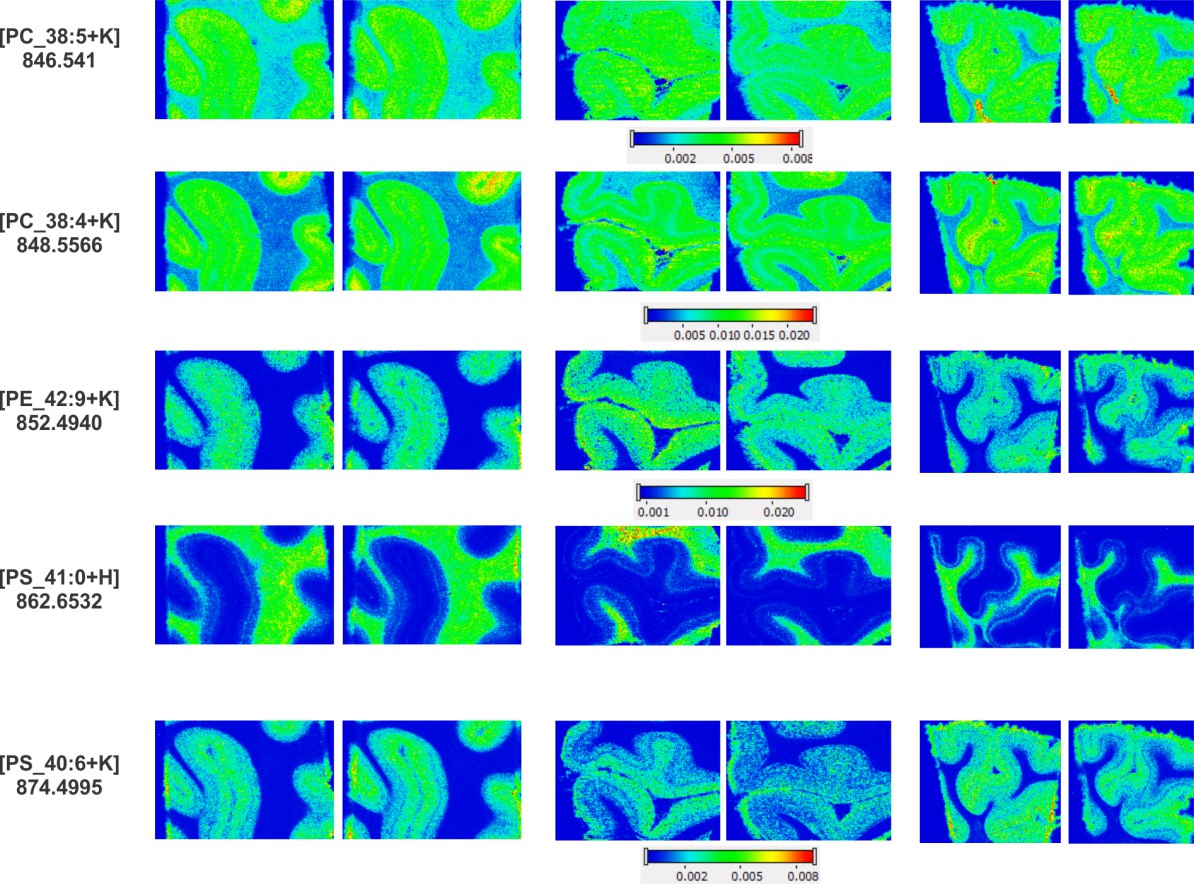


**Figure S16.** Anatomical distribution of glycerophospholipids in the primary visual cortex of three human brains.


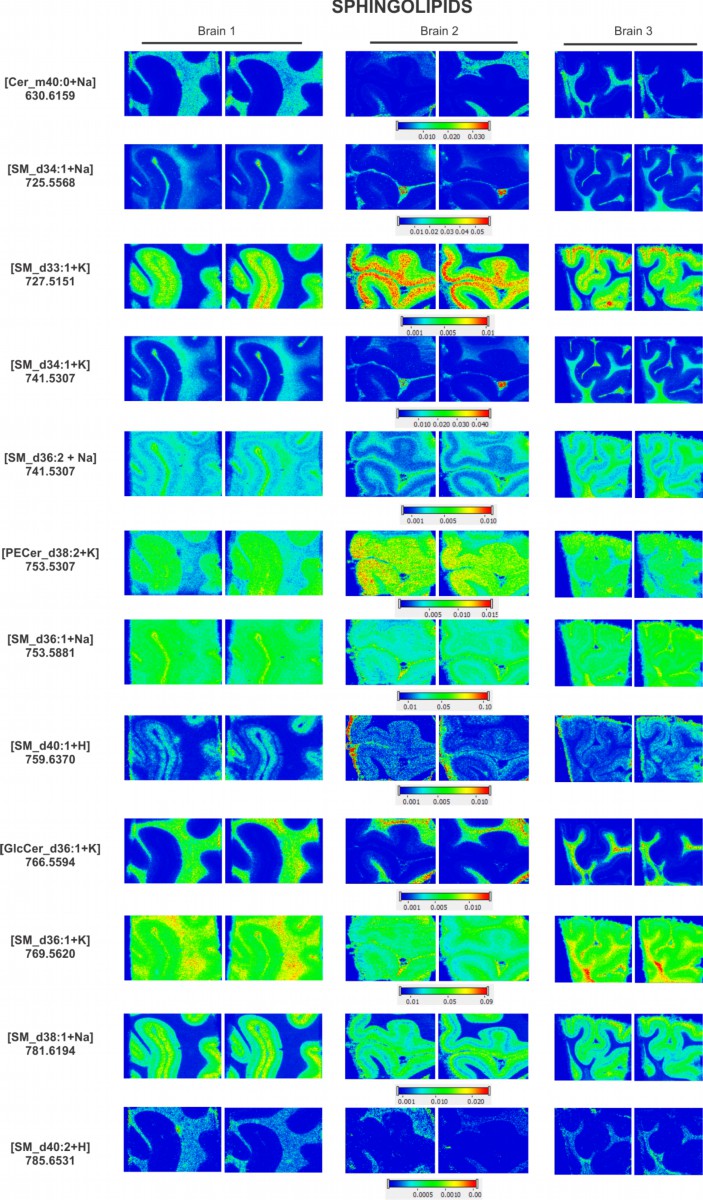


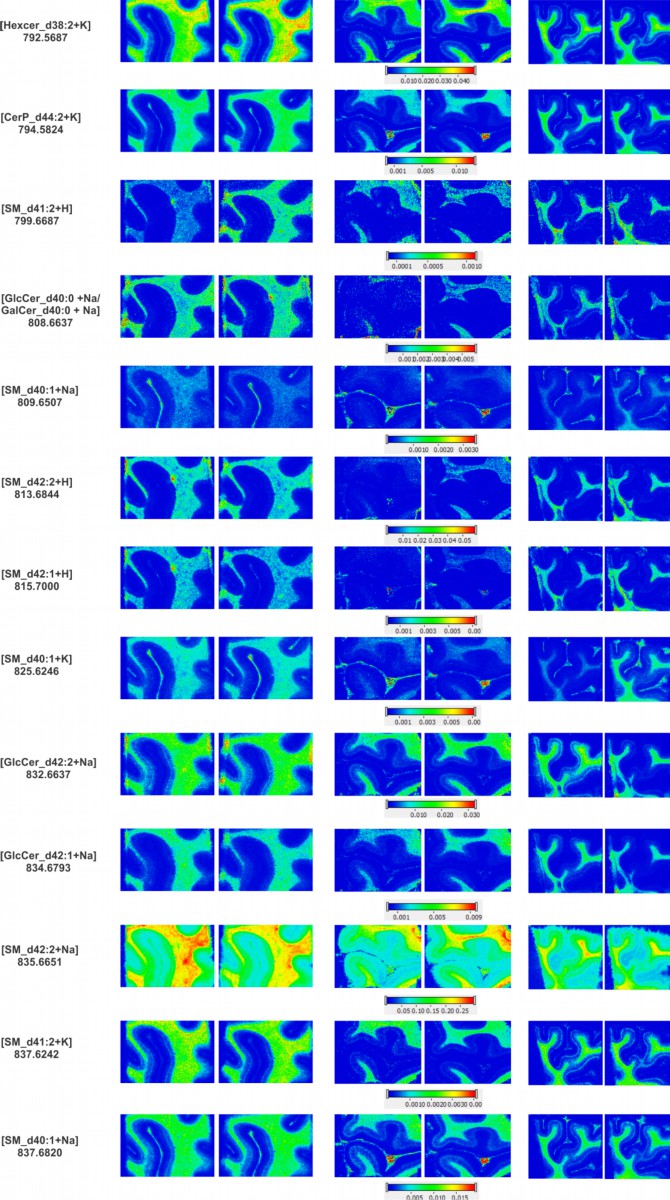


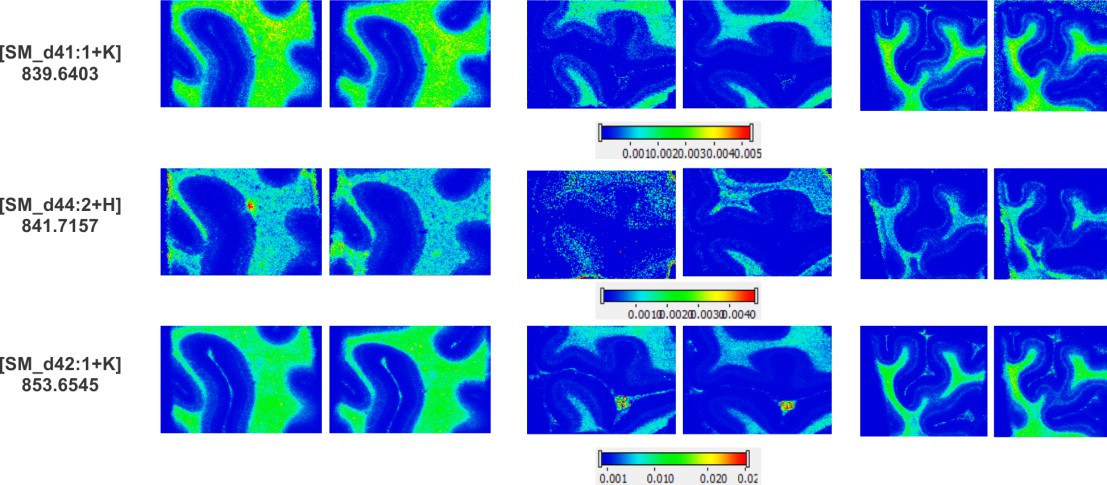


**Figure S17.** Anatomical distribution of sphingolipids in the primary visual cortex of three human brains.


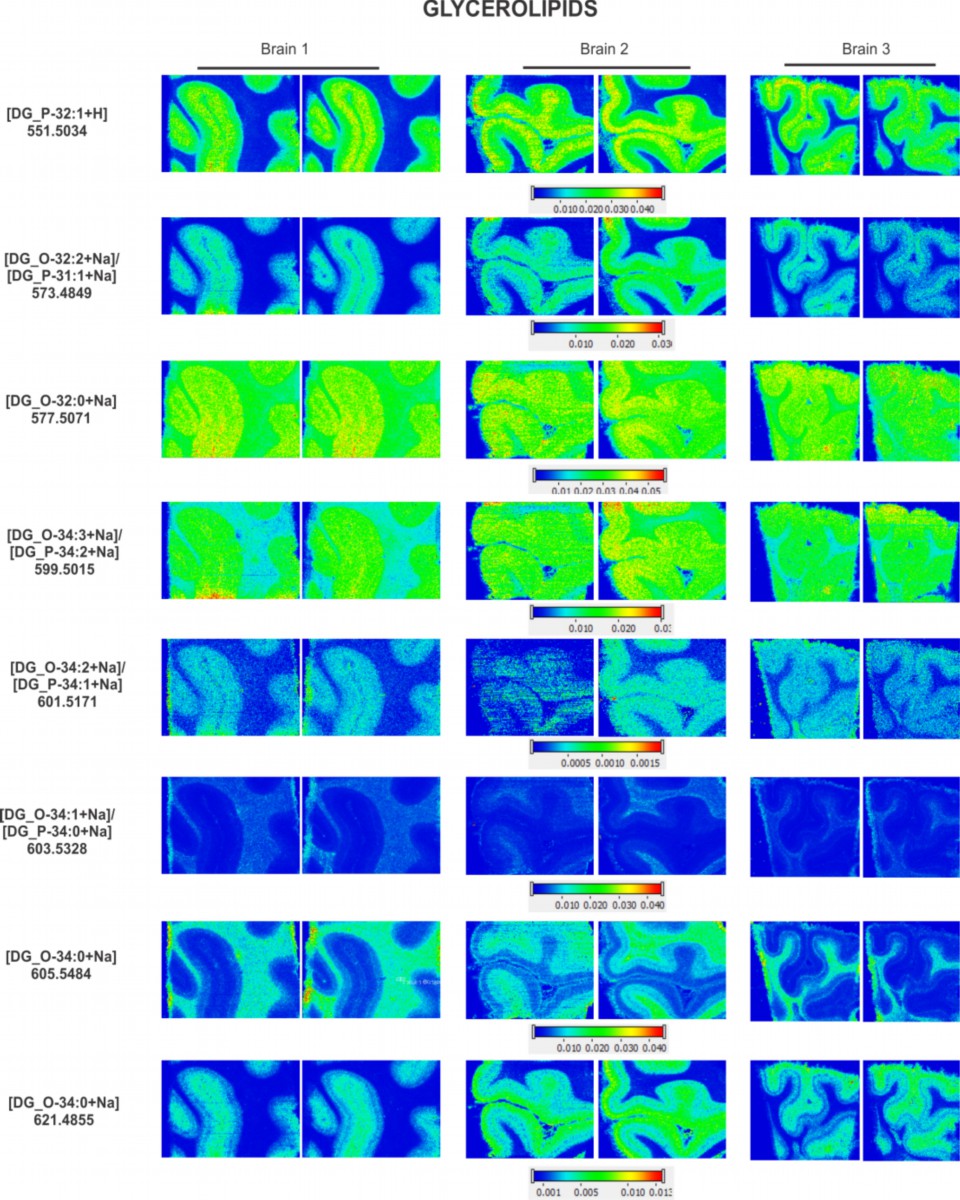


**Figure S18.** Anatomical distribution of glycerolipids in the primary visual cortex of three human brains.
